# Dynamic analysis and optimal control of a stochastic investor sentiment contagion model considering sentiments isolation with random parametric perturbations

**DOI:** 10.1038/s41598-023-48575-7

**Published:** 2023-12-02

**Authors:** Sida Kang, Xilin Hou, Yuhan Hu, Hongyu Liu

**Affiliations:** 1 School of Electronic and Information Engineering, University of Science and Technology Liaoning, Anshan, 114051 China; 2 School of Science, University of Science and Technology Liaoning, Anshan, 114051 China; 3 School of Business Administration, University of Science and Technology Liaoning, Anshan, 114051 China

**Keywords:** Applied mathematics, Nonlinear phenomena

## Abstract

Investor sentiment contagion has a profound influence on economic and social development. This paper explores the diverse influences of various investor sentiments in modern society on the economy and society. It also investigates the interference of various uncertain factors on investor sentiments in the modern economy and society. On this basis, the dual-system stochastic *SPA*2*G*2*R* model was constructed, incorporating positive and negative sentiments, as well as a supervision and isolation mechanism. The global existence of positive solutions was established, and sufficient conditions for the disappearance and steady distribution of investor sentiment were calculated. An optimal control strategy for the stochastic model was put forward, with numerical simulation supporting the theoretical analysis results. A comparison with parameter changes in the deterministic model was also conducted. The research reveals a competitive relationship between different investor sentiments. Enhancing societal guidance mechanisms promotes positive investor sentiment contagion. Timely control by the supervisory department effectively curbs the spread of investor sentiment. Additionally, white noise promotes investor sentiment contagion, suggesting effective regulation through control of noise intensity and disturbance parameters.

## Introduction

The production, contagion, and spread of investor sentiment have played an indispensable role in the development of human economic activities. Generally, investors express positive and negative sentiments during different stages of investor sentiment contagion in the development of the market economy^1^. At the same time, given the constant changes in social demand, investor sentiments of various natures require timely macro-control to adapt to the times^2^. Therefore, studying the contagion mechanism and control measures of investor sentiment is crucial.

The mechanism of investor sentiment contagion bears a striking resemblance to that of infectious diseases and information transmission^3,4^. Therefore, scholars usually study investor sentiment contagion based on classical models of infectious diseases and information transmission, such as the *SI* model^5^, the *SIS* model^6^, and the *ILSR* model^7^. Subsequently, a series of models were successively put forward, including the *SIR* sentiment contagion model with an interactive mechanism^8^, the $SEI_1I_2R$ sentiment contagion model with different group characteristics^9^, the $HAR-RV$ sentiment contagion model with media report effect^10^, and the $MNE-SFI$ sentiment contagion model with dynamic multiple mechanisms^11^.

In recent years, scholars have conducted extensive studies on the influence of investor sentiment on the economy and the market. Naeem et al.^12^ tested the predictive abilities of online investors for six major cryptocurrency returns. Their study shows that online investor sentiment is an important non-linear predictor of most major cryptocurrency returns. Jing et al.^13^ proposed a model combining deep learning and sentiment analysis to predict share prices. Gong et al.^14^ introduced an investor sentiment index based on partial ordinary least squares techniques, enabling the predictability of stock volatility through sentiment measures. Wang et al.^15^ comprehensively studied the causal relationship between the crude oil futures market and investor sentiment under extreme impacts. The results indicated that crude oil futures were more susceptible to negative extreme impacts than positive ones. Chen et al.^16^ revealed the predictability of the energy futures market involving investor sentiment. They introduced a new investor sentiment index capturing the characteristics of the energy futures market, including sentiment conversion and internet attention. Ho^17^ analyzed the non-linear causality between crude oil prices and Chinese investor sentiment, considering time-varying effects and dynamic influences. The research results show that oil prices have a time-varying negative effect on Chinese investor sentiment in most cases. Piñeiro-Chousa^18^ used panel data to analyze the influence of investor sentiment extracted from social networks on the green bonds market. According to recent research results, most scholars concur that investor sentiment has the most prominent influence on the stock market^19,20^.

Meanwhile, the study on investor sentiment contagion has gradually become a research hotspot in recent years. Han et al.^21^ proposed a set of compound methods based on wavelet, contagion entropy, and network analysis to explore the model of investor sentiment contagion among enterprises. In an effort to elucidate the influence of investor sentiment on the stock market, Chen et al.^22^ constructed the dynamic *SIRS* model based on the integration of investor sentiment, investor structure, and the capital market. The research results demonstrate that as the influence of investors’ mutual communication increases or the calm sentiment rate decreases, investor sentiment will begin to spread, leading to an increased probability of frenzied overbought conditions in the stock market. Song et al.^23^ and Liu et al.^24^ constructed the $SOSa-SPSa$ sentiment contagion model, considering both optimism and pessimism and discussed the model’s application in finance.

On this basis, the research on uncertainty AI methods for uncertainty data has also widely concerned in recent years. This also provides theoretical and methodological support for the study of the disturbance of uncertainty factors on the investor sentiment contagion. Wang^25^ propose a bottom-up layer-by-layer design scheme, using the Wang-Mendel method (WM Method) to design each layer of fuzzy systems and a DCFS with parameter sharing to save memory and computational resources. And then apply the DCFS model to predict a synthetic chaotic plus random time series and the Hang Seng Index of the Hong Kong stock market. Chen et al.^26^ found that the granular mean shift clustering algorithm has better clustering performance than traditional clustering algorithms, such as Kmeans, Gaussian mixture, etc. Sang et al.^27^ proposed a fuzzy rough feature selection method based on robust non-linear vague quantifier for ordinal classification. Tong et al.^28^ proposed a finite-time adaptive fuzzy event-triggered output-feedback control design method under the framework of finite-time stability criterion and adaptive backstepping control design technique, and rigorously proved the semi-global finite-time stability of the control system. He et al.^29^ proposed a granular elastic network regression model based on granules to solve the problem of traditional linear regression models that are difficult to handle uncertain data. They found that granular elastic network has better fitting advantage than traditional linear regression model.

The aforementioned scholars made extensive studies on the influences of investor sentiment on different economies and markets. However, there are relatively few studies on the dynamic process of investor sentiment contagion. In addition, most studies on investor sentiment contagion are concentrated in deterministic environments. These studies ignore the interference of random factors on the contagion of investor sentiment. Normally, the realistic social system is complex, with many uncertain factors^30^, and the factors influencing investor sentiment are often random. And the studies that include a stochastic perturbation term in deterministic investor sentiment contagion models are also uncommon. At the same time, positive investor sentiment tends to foster development in the economy and society, while negative investor sentiment usually restricts economic and social progress^31–33^. Supervisors could find it more beneficial to control investor sentiment by supervising different investor sentiments and isolating the disseminators of investor sentiment to adapt to various social demands better. Unlike the isolation of disease spread, regulatory isolation of investor sentiment contagion only requires disseminators to refrain from expressing their views. On this basis, this paper puts forward the stochastic *SPA*2*G*2*R* model, considering various investor sentiment contagions and regulatory isolation. The uniqueness of the global existence of positive solutions is established. After calculating the sufficient conditions of information disappearance and steady information distribution, appropriate parameters are selected as control variables. Finally, numerical simulation is employed to verify the rationality of the proposed theorem.

The remaining sections are arranged as follows. In “The model”, the stochastic *SPA*2*G*2*R* model considering different investor sentiment contagions and regulatory isolation is constructed. “Existence of the global and positive solution” proves the uniqueness of the global existence of positive solutions. “Disappearance of the Information” gives sufficient conditions for investor sentiment disappearance. “A sufficient condition for the stationary distribution” gives sufficient conditions for the steady distribution of investor sentiment. “The stochastic optimal control model” introduces the optimal control existence and optimal control strategy for different investor sentiment contagions, as well as the supervision and isolation. In “Numerical simulations”, numerical simulation is used to analyze the influence of random disturbance strength on investor sentiment contagion as well as supervision and isolation. The last section gives conclusions.

## The model

**Figure 1:**
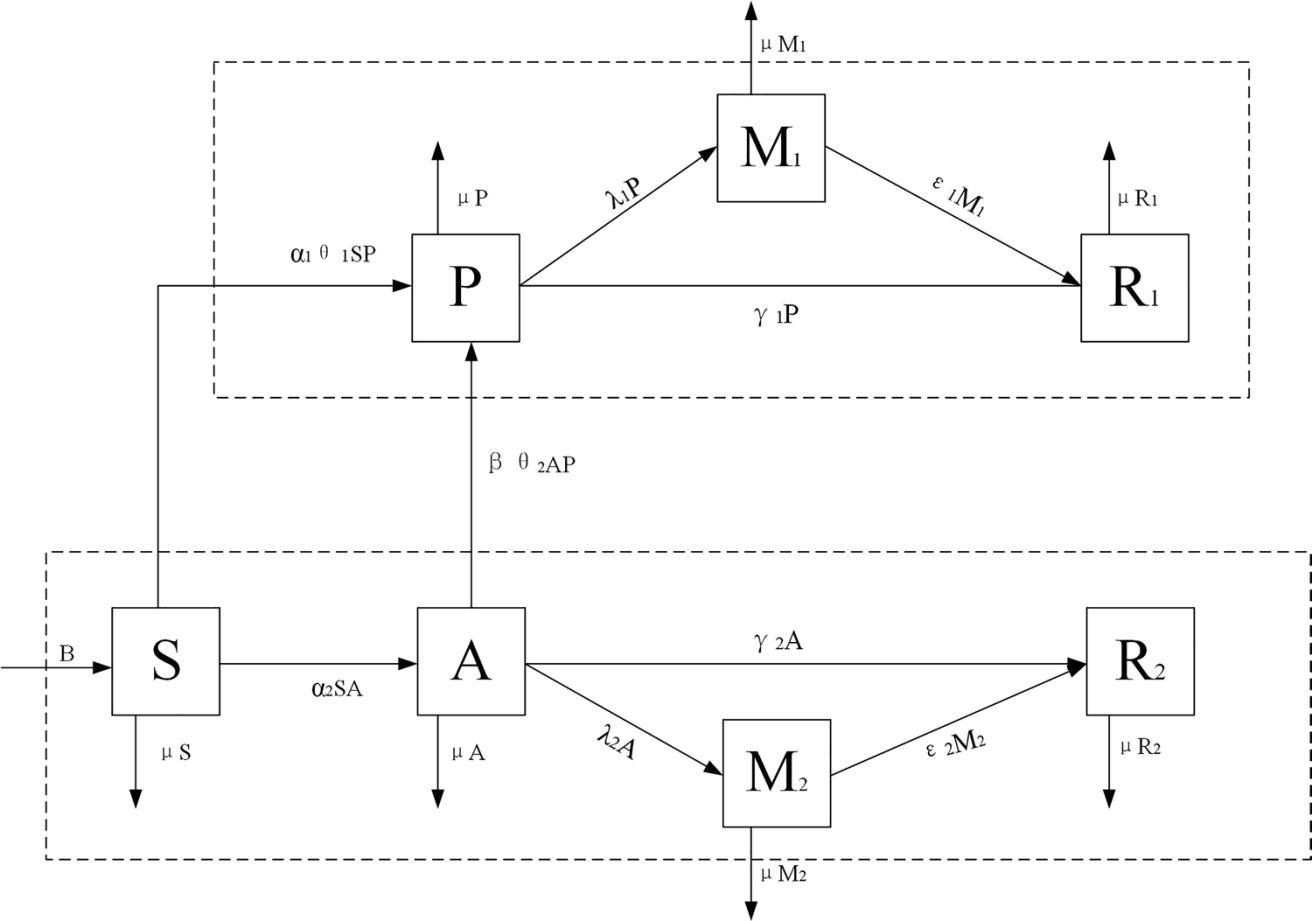
The flow diagram of the model.

This study considers an open virtual community where the population size changes with time *t*. The total population size can be expressed by *N*(*t*). Individuals in the community are categorized as follows: (1) Susceptible individuals who have not been exposed to any type of investor sentiment, *S*(*t*); (2) Disseminators of positive investor sentiment, *P*(*t*); (3) Disseminators of negative investor sentiment, *A*(*t*); (4) Individuals under supervision and isolation from disseminators of positive and negative investor sentiments, $G_1(t)$ and $G_2(t)$, respectively. (5) Individuals who no longer disseminate positive or negative investor sentiment, $R_1(t)$ and $R_2(t)$, respectively. According to the meanings represented by each compartment, and the flow relationships between them, a flow diagram of the model can be constructed, as shown in Fig. 1.

Based on Fig. 1, a *SPA*2*G*2*R* model can be constructed. The parameters in Fig. 1 can be interpreted as follows:

- The number of individuals in the social system generally changes with time. Therefore, this paper defines *B* as the number of people who enter the social system. $\mu$ is defined as the rate of individuals moving out of the social system due to force majeure;
- As positive and negative investor sentiments begin to disseminate in the social system, susceptible individuals will have a probability of coming into contact with disseminators of investor sentiments. Therefore, the rate of contact with disseminators of positive investor sentiment is defined as $\alpha _1$, and the rate of contact with disseminators of negative investor sentiment is defined as $\alpha _2$. Simultaneously, susceptible individuals have a certain probability $\theta _1$ of being influenced by the guidance mechanism and consequently becoming disseminators of positive investor sentiment;
- When positive and negative investor sentiments are simultaneously disseminated in the social system, there exists a probability that disseminators of these two sentiments come into contact with each other. Therefore, this mutual contact rate of disseminators of the two investor sentiments is defined as $\beta$. Similarly, disseminators of negative investor sentiment have a probability $\theta _2$ of being influenced by guidance mechanisms, such as self-learning or publicity, and thus become disseminators of positive investor sentiment;
- When the social system deems it unnecessary for the two types of investor sentiments, some disseminators of investor sentiment have certain probabilities $\gamma _1$ and $\gamma _2$ to actively choose to cease investor sentiment contagion due to the effectiveness of information. Other disseminators of investor sentiment have probabilities $\lambda _1$ and $\lambda _2$ of undergoing regulatory isolation by the management, transforming into isolated groups $G_1$ and $G_2$ of investor sentiment. In addition, as the disseminated investor sentiments cease to spread, the isolated groups of investor sentiment experience a reduction in the enthusiasm for investor sentiment contagion. Finally, they have probabilities $\epsilon _1$ and $\epsilon _2$ of choosing not to disseminate investor sentiment any longer.

In addition, the uncertain factors in social systems are commonly referred to as environmental noise. It is not scientific to study the spread of investor sentiment while ignoring random environmental noise fluctuations. Incorporating environmental noise into deterministic models is more representative of how investor sentiment contagion in real society. The random factors added to the spread models mainly include three classical approaches: (1) Introducing Gaussian white noise into deterministic parameter perturbation models^34^. (2) Random perturbation encompassing the positive endemic equilibrium of deterministic models^35^. (3) Alternating between regimes based on the probability of Markov chains^36^. Since random perturbations in the environment may affect the contact rate under guidance mechanism and the proportion of investor sentiment disseminators under regulatory quarantine, this paper uses Gaussian white noise to generate random perturbations of $\theta _1$, $\theta _2$, $\lambda _1$ and $\lambda _2$, and the parameters of random perturbation are expressed as follows:

1 $$\begin{aligned} \ {\theta _1} \rightarrow {\theta _1} + {\sigma _1}{\dot{W}_1}(t),{\theta _2} \rightarrow {\theta _2} + {\sigma _2}{\dot{W}_2}(t),{\lambda _1} \rightarrow {\lambda _1} + {\sigma _3}{\dot{W}_3}(t),{\lambda _2} \rightarrow {\lambda _2} + {\sigma _4}{\dot{W}_4}(t). \end{aligned}$$

Here, $W_i(i=1,2,3,4)$ are independent standard Brownian motions and $\sigma _i^2 > 0(i=1,2,3,4)$ represent the intensities of $W_i(i=1,2,3,4)$, respectively. In this paper, $W_1$, $W_2$, $W_3$ and $W_4$ represent the relationship without mutual influence between $\theta _1$, $\theta _2$, $\lambda _1$ and $\lambda _2$ , respectively.

The stochastic perturbation parameters are introduced into the deterministic model to construct a stochastic *SPA*2*G*2*R* model driven by Gaussian white noise, and the stochastic model can be represented as:

2 $$\begin{aligned} \begin{aligned} \left\{ {\begin{array}{*{20}{l}} {dS(t)}&{} = &{} {\left( {B - {\alpha _1}{\theta _1}SP - {\alpha _2}SA - \mu S} \right) dt - {\alpha _1}{\sigma _1}SPd{W_1}(t),}\\ {dP(t)}&{} = &{}\begin{array}{l} \left( {{\alpha _1}{\theta _1}SP + \beta {\theta _2}AP - {\lambda _1}P - {\gamma _1}P - \mu P} \right) dt\\ + {\alpha _1}{\sigma _1}SPd{W_1}(t) + \beta {\sigma _2}APd{W_2}(t) - {\sigma _3}Pd{W_3}(t), \end{array}\\ {dA(t)}&{} = &{}\begin{array}{l} \left( {{\alpha _2}SA - \beta {\theta _2}AP - {\lambda _2}A - {\gamma _2}A - \mu A} \right) dt\\ - \beta {\sigma _2}APd{W_2}(t) - {\sigma _4}Ad{W_4}(t), \end{array}\\ {d{G_1}(t)}&{} = &{}{\left( {{\lambda _1}P - {\varepsilon _1}{G_1} - \mu {G_1}} \right) dt + {\sigma _3}Pd{W_3}(t),}\\ {d{G_2}(t)}&{} = &{}{\left( {{\lambda _2}A - {\varepsilon _2}{G_2} - \mu {G_2}} \right) dt + {\sigma _4}Ad{W_4}(t).} \end{array}} \right. \end{aligned} \end{aligned}$$

## Existence of the global and positive solution

In the rest of this paper, let $(\Omega ,{{\mathscr {F}}},{\{ {{\mathscr {F}}}{_t}\} _{t \ge 0}},P)$ be a complete probability space with a filtration ${\{ {{\mathscr {F}}}{_t}\} _{t \ge 0}}$ satisfying the usual conditions. And while ${{\mathscr {F}}}{_0}$ contains all $P-null$ sets, it is increasing and right continuous^37^. It also can be denoted as:

3 $$\begin{aligned} \begin{aligned} \ {\mathbb {R}}_ + ^5 = \{ ({x_1},{x_2},{x_3},{x_4},{x_5})\mathrm{{|}}{x_i} > 0,i = 1,2,3,4,5\}.\ \end{aligned} \end{aligned}$$

Whether the global solution is existence is the basis of analyzing the dynamic behavior of stochastic system (2). At the same time, according to the actual situation, it is required a positive value for the dynamic model of investor sentiment contagion. The stochastic system (2) can be proved global and positive by Theorem 1.

### Theorem 1

The existence of a unique positive solution $({S}(t),{P}(t),{A}(t),{G_1}(t),{G_2}(t)) \in {\mathbb {R}}_ + ^5$ of stochastic system (2) is satisfied any given initial value $({S}(t),{P}(t),{A}(t),{G_1}(t),{G_2}(t)) \in {\mathbb {R}}_ + ^5$. The probability of the solution is 1 and remains in ${\mathbb {R}}_ + ^5$.

### Proof

The existence of a unique local positive solution $({S}(t),{P}(t),{A}(t),{G_1}(t),{G_2}(t)) \in {\mathbb {R}}_ + ^5$ of stochastic system (2) on $t \in [0,{\tau _e})$, which is based on the coefficients of deterministic system are locally Lipschitz continuous of any given initial value $({S}(t),{P}(t),{A}(t),{G_1}(t),{G_2}(t)) \in {\mathbb {R}}_ + ^5$. ${\tau _e}$ is the explosion time^38^. It is need to have that ${\tau _e} = \infty$ a.s. to show this solution globally. The stopping time ${\tau ^ + }$ can be defined by:

4 $$\begin{aligned} \begin{aligned} \ {\tau ^ + } = \inf \left\{ {t \in [0,{\tau _e}):S(t) \ge 0\mathrm{{ }}or\mathrm{{ }}P(t) \ge 0\mathrm{{ }}or\mathrm{{ }}A(t) \ge 0or{\mathrm{{G}}_1}(t) \ge 0\mathrm{{ }}or\mathrm{{ }}{\mathrm{{G}}_2}(t) \ge 0\mathrm{{ }}} \right\} .\ \end{aligned} \end{aligned}$$

Let set $\inf \emptyset = \infty$ ($\emptyset$ denotes the empty set). It is easy to get ${\tau ^ + } \le {\tau _e}$. So if ${\tau ^ + } = \infty$ a.s. is proved, then ${\tau _e} = \infty$ and $({S}(t),{P}(t),{A}(t),{G_1}(t),{G_2}(t)) \in {\mathbb {R}}_ + ^5$ a.s. for all $t \ge 0$. Assume that ${\tau ^ + } < \infty$, then $T>0$ is existence such that $P({\tau ^ + } < T) > 0$. Define ${\mathcal {C}}^2$ function *V*: ${\mathbb {R}}_ + ^5 \rightarrow {\mathbb {R}}_ + ^5$ by $V(X) = In\mathrm{{ }}SPA{G_1}{G_2}$. Let using $It{\hat{o}}'s$ formula to calculate the differential of *V* along the solution trajectories of stochastic system (2). For $\omega \in ({\tau ^ + } < T)$ and for all $t \in [0,{\tau _e})$, we get

5 $$\begin{aligned} \begin{aligned} \ \begin{array}{*{20}{l}} {dV(X(t))}&{} = &{}{\left[ {\frac{B}{S} - {\alpha _1}{\theta _1}P - {\alpha _2}A - \mu - \frac{1}{2}\alpha _1^2\sigma _1^2{P^2}} \right] dt + \left[ \begin{array}{l} {\alpha _1}{\theta _1}S + \beta {\theta _2}A - {\lambda _1} - {\gamma _1} - \mu \\ - \frac{1}{2}\alpha _1^2\sigma _1^2{S^2} - \frac{1}{2}{\beta ^2}\sigma _2^2{A^2} - \frac{1}{2}\sigma _3^2 \end{array} \right] dt}\\ {}&{}{}&{}{ + \left[ \begin{array}{l} {\alpha _2}S - \beta {\theta _2}P - {\lambda _2} - {\gamma _2} - \mu \\ - \frac{1}{2}{\beta ^2}\sigma _2^2{P^2} - \frac{1}{2}\sigma _4^2 \end{array} \right] dt + \left[ {\frac{{{\lambda _1}P}}{{{G_1}}} - {\varepsilon _1} - \mu - \frac{1}{2}\sigma _3^2\frac{{{P^2}}}{{G_1^2}}} \right] dt}\\ {}&{}{}&{}{ + \left[ {\frac{{{\lambda _2}A}}{{{G_2}}} - {\varepsilon _2} - \mu - \frac{1}{2}\sigma _4^2\frac{{{A^2}}}{{G_2^2}}} \right] dt - {\alpha _1}{\sigma _1}Pd{W_1} + {\alpha _1}{\sigma _1}Sd{W_1} + \beta {\sigma _2}Ad{W_2} - {\sigma _3}d{W_3}}\\ {}&{}{}&{}{ - \beta {\sigma _2}Pd{W_2} - {\sigma _4}d{W_4} + \frac{{{\sigma _3}P}}{{{G_1}}}d{W_3} + \frac{{{\sigma _4}A}}{{{G_2}}}d{W_4}.} \end{array}\ \end{aligned} \end{aligned}$$

Positivity of *X*(*t*) implies that

6 $$\begin{aligned} \begin{aligned} \ \begin{array}{*{20}{l}} {dV(X(t))}&{} \ge &{}{L(S,P,A,{G_1},{G_2})dt - {\alpha _1}{\sigma _1}(P - S)d{W_1} + \beta {\sigma _2}(A - P)d{W_2}}\\ {}&{}{}&{}{ - {\sigma _3}(1 - \frac{P}{{{G_1}}})d{W_3} - {\sigma _4}(1 - \frac{A}{{{G_2}}})d{W_4},} \end{array}\ \end{aligned} \end{aligned}$$

where

7 $$\begin{aligned} \begin{aligned} \ \begin{array}{*{20}{l}} {L(S,P,A,{G_1},{G_2})}&{} = &{}{ -\, \mu - ({\lambda _1} + {\gamma _1} + \mu ) - ({\lambda _2} + {\gamma _2} + \mu ) - ({\varepsilon _1} + \mu ) - ({\varepsilon _2} + \mu )}\\ {}&{}{}&{}{ - \,\frac{1}{2}\alpha _1^2\sigma _1^2{P^2} - \frac{1}{2}\alpha _1^2\sigma _1^2{S^2} - \frac{1}{2}{\beta ^2}\sigma _2^2{A^2} - \frac{1}{2}\sigma _3^2 - \frac{1}{2}\sigma _4^2}\\ {}&{}{}&{}{ -\, \frac{1}{2}{\beta ^2}\sigma _2^2{P^2} - \frac{1}{2}\sigma _3^2\frac{{{P^2}}}{{G_1^2}} - \frac{1}{2}\sigma _4^2\frac{{{A^2}}}{{G_2^2}}.} \end{array}\ \end{aligned} \end{aligned}$$

So we have

8 $$\begin{aligned} \begin{aligned} \ \begin{array}{*{20}{l}} {V(X(t))}&{} \ge &{}{V({X_0}) + \int _0^t {L(S(u),P(u),A(u),{G_1}(u),{G_2}(u))du} }\\ {}&{}{}&{}{ - \int _0^t {{\alpha _1}{\sigma _1}(p(u) - S(u))d{W_1}(u)} - \int _0^t {\beta {\sigma _2}(A(u) - P(u))d{W_2}(u)} }\\ {}&{}{}&{}{ - \int _0^t {{\sigma _3}(1 - \frac{{P(u)}}{{{G_1}(u)}})d{W_3}(u)} - \int _0^t {{\sigma _4}(1 - \frac{{A(u)}}{{{G_2}(u)}})d{W_4}(u)}.} \end{array}\ \end{aligned} \end{aligned}$$

Note that some components of $X \left( \tau ^+ \right)$ equal 0. Thereby

9 $$\begin{aligned} \begin{aligned} \ \mathop {\lim }\limits _{t \rightarrow {\tau ^ + }} V(X(t)) = - \infty .\ \end{aligned} \end{aligned}$$

Letting $t \rightarrow {\tau ^ + }$ in system (8), one have

10 $$\begin{aligned} \begin{aligned} \ \begin{array}{*{20}{l}} { - \infty }&{} \ge &{}{V({X_0}) + \int _0^{{\tau ^ + }} {L(S(u),P(u),A(u),{G_1}(u),{G_2}(u))du} }\\ {}&{}{}&{}{ - \int _0^{{\tau ^ + }} {{\alpha _1}{\sigma _1}(p(u) - S(u))d{W_1}(u)} - \int _0^{{\tau ^ + }} {\beta {\sigma _2}(A(u) - P(u))d{W_2}(u)} }\\ {}&{}{}&{}{ - \int _0^{{\tau ^ + }} {{\sigma _3}(1 - \frac{{P(u)}}{{{G_1}(u)}})d{W_3}(u)} - \int _0^{{\tau ^ + }} {{\sigma _4}(1 - \frac{{A(u)}}{{{G_2}(u)}})d{W_4}(u)} > - \infty .} \end{array}\ \end{aligned} \end{aligned}$$

According to Eq. (8) and Eq. (9), it can be obtained that Eq. (10) is less than or equal to $$-\infty $$. Meanwhile, for any given initial value $$({S}(0),{P}(0),{A}(0),{G_1}(0),{G_2}(0)) \in {\mathbb {R}}_ + ^5$$ and $$S(u),P(u),A(u),G_1(u),G_2(u)$$ in Eq. (10) belong to a positive invariant set and is bounded. Therefore, $$S(u),P(u),A(u),G_1(u),G_2(u)$$ are greater than 0 and greater than $$-\infty $$, then Eq. (10) is greater than $$-\infty $$. This result is contradictory. In addition, the result obtained by Eq. (10) rejects the original hypothesis $${\tau ^ + } < \infty 
$$. Thus, $$\tau ^+=\infty $$. $$\square $$

## Disappearance of the information

Theorem 2 and Theorem 3 give the condition for the disappearance of the investor sentiment. The condition is expressed by intensities of noises and parameters of deterministic system. In the stochastic *SPA*2*G*2*R* model built in this paper, (1) Theorem 2 gives the condition for the disappearance of positive investor sentiment, (2) Theorem 3 gives the condition for the disappearance of negative investor sentiment.

### Theorem 2

For any given initial value $({S}(0),{P}(0),{A}(0),{G_1}(0),{G_2}(0)) \in {\mathbb {R}}_ + ^5$, $\mathop {\lim }\limits _{t \rightarrow \infty } \sup \frac{{\ln P(t)}}{t} \le K(\sigma _1^2,\sigma _2^2,\sigma _3^2)$ holds a.s.. Further, $K(\sigma _1^2,\sigma _2^2,\sigma _3^2)<0$, then *P*(*t*) tend to 0 exponentially a.s., where $K \left( \sigma _1^2,\sigma _2^2,\sigma _3^2 \right) = \frac{{\theta _1^2}}{{2\sigma _1^2}} + \frac{{\theta _2^2}}{{2\sigma _2^2}} - ({\lambda _1} + {\gamma _1} + \mu + \frac{1}{2}\sigma _3^2)$.

### Proof

Use $It{\hat{o}}'s$ formula to calculate the differentiation of *P*(*t*) in stochastic system (2), and $d\ln P(t)$ can be written as:

11 $$\begin{aligned} \begin{aligned} \ \begin{array}{*{20}{l}} {d\ln P(t)}&{} = &{}{\left[ {{\alpha _1}{\theta _1}S + \beta {\theta _2}A - ({\lambda _1} + {\gamma _1} + \mu ) - \frac{1}{2}\alpha _1^2\sigma _1^2{S^2} - \frac{1}{2}{\beta ^2}\sigma _2^2{A^2} - \frac{1}{2}\sigma _3^2} \right] dt}\\ {}&{}{}&{}{ + {\alpha _1}{\sigma _1}Sd{W_1} + \beta {\sigma _2}Ad{W_2} - {\sigma _3}d{W_3}.} \end{array}\ \end{aligned} \end{aligned}$$

Thus, $\ln {P}(t)$ can be denoted as:

12 $$\begin{aligned} \begin{aligned} \ \begin{array}{*{20}{l}} {\ln P(t)}&{} = &{}{\ln P(0) + \int _0^t {\left[ \begin{array}{l} {\alpha _1}{\theta _1}S(u) + \beta {\theta _2}A(u) - ({\lambda _1} + {\gamma _1} + \mu )\\ - \frac{1}{2}\alpha _1^2\sigma _1^2{S^2}(u) - \frac{1}{2}{\beta ^2}\sigma _2^2{A^2}(u) - \frac{1}{2}\sigma _3^2 \end{array} \right] du} }\\ {}&{}{}&{}{ + \int _0^t {{\alpha _1}{\sigma _1}S(u)d{W_1}(u)} + \int _0^t {\beta {\sigma _2}A(u)d{W_2}(u)} - {\sigma _3}d{W_3}(t).} \end{array}\ \end{aligned} \end{aligned}$$

Denote

13 $$\begin{aligned} \begin{aligned} \ \begin{array}{l} {\Phi _1}(t) = \int _0^t {{\alpha _1}{\sigma _1}S(u)d{W_1}(u)},\\ {\Phi _2}(t) = \int _0^t {\beta {\sigma _2}A(u)d{W_2}(u)}, \end{array}\ \end{aligned} \end{aligned}$$

${\Phi _1}(t)$ and ${\Phi _2}(t)$ are continuous local martingale. The quadratic variation of ${\Phi _1}(t)$ and ${\Phi _2}(t)$ can be denoted as:

14 $$\begin{aligned} \begin{aligned} \ \begin{array}{l} \left\langle {{\Phi _1}(t)} \right\rangle = \sigma _1^2\int _0^t {\alpha _1^2{S^2}(u)du},\\ \left\langle {{\Phi _2}(t)} \right\rangle = \sigma _2^2\int _0^t {{\beta ^2}{A^2}(u)du}. \end{array}\ \end{aligned} \end{aligned}$$

By exponential martingale inequality^38^, it can be known that

15 $$\begin{aligned} \begin{aligned} \ P\left\{ {\mathop {\sup }\limits _{0 \le t \le k} [\Phi (t) - \frac{c}{2}\left\langle {\Phi (t)} \right\rangle ] > \frac{2}{c}\ln k} \right\} \le {k^{ - \frac{2}{c}}},\ \end{aligned} \end{aligned}$$

where $0<c<1$, *k* is a random integer. Using Borel-Cantelli lemma, it is easy to know that the random integer $k_0(\omega )$ exists such that for $k>k_0$ for almost all $\omega \in \Omega$, ${\sup _{0 \le t \le k}}[\Phi (t) - \frac{c}{2}\left\langle {\Phi (t)} \right\rangle ] \le \frac{2}{c}$. Therefore, for all $t \in [0,k]$, one have

16 $$\begin{aligned} \begin{aligned} \ \begin{array}{l} \int _0^t {{\alpha _1}{\sigma _1}S(u)d{W_1}(u) \le \frac{1}{2}} c\sigma _1^2\int _0^t {\alpha _1^2{S^2}(u)du} + \frac{2}{c}\ln k,\\ \int _0^t {\beta {\sigma _2}A(u)d{W_2}(u) \le \frac{1}{2}} c\sigma _2^2\int _0^t {{\beta ^2}{A^2}(u)du} + \frac{2}{c}\ln k. \end{array}\ \end{aligned} \end{aligned}$$

Then, it can be obtained that

17 $$\begin{aligned} \begin{aligned} \ \begin{array}{*{20}{l}} {\ln P(t)}&{} \le &{}{\ln P(0) + \int _0^t {\left[ \begin{array}{l} {\alpha _1}{\theta _1}S(u) + \beta {\theta _2}A(u) - ({\lambda _1} + {\gamma _1} + \mu ) - \frac{1}{2}\sigma _3^2\\ - \frac{1}{2}(1 - c)\alpha _1^2\sigma _1^2{S^2}(u) - \frac{1}{2}(1 - c){\beta ^2}\sigma _2^2{A^2}(u) \end{array} \right] du} }\\ {}&{}{}&{}{ + \frac{2}{c}\ln k + \frac{2}{c}\ln k - {\sigma _3}{W_3}(t),} \end{array}\ \end{aligned} \end{aligned}$$

noting that

18 $$\begin{aligned} \begin{aligned} \ \begin{array}{l} {\alpha _1}{\theta _1}S(u) - \frac{1}{2}(1 - c)\alpha _1^2\sigma _1^2{S^2}(u) \le \frac{{\theta _1^2}}{{2(1 - c)\sigma _1^2}},\\ \beta {\theta _2}A(u) - \frac{1}{2}(1 - c){\beta ^2}\sigma _2^2{A^2}(u) \le \frac{{\theta _2^2}}{{2(1 - c)\sigma _2^2}}. \end{array}\ \end{aligned} \end{aligned}$$

Substituting Eq. (18) into Eq. (17), $\ln P(t)$ can be written as:

19 $$\begin{aligned} \begin{aligned} \ \begin{array}{*{20}{l}} {\ln P(t)}&{} \le &{}{\ln P(0) + \int _0^t {\left[ {\frac{{\theta _1^2}}{{2(1 - c)\sigma _1^2}} + \frac{{\theta _2^2}}{{2(1 - c)\sigma _2^2}} - ({\lambda _1} + {\gamma _1} + \mu + \frac{1}{2}\sigma _3^2)} \right] du} }\\ {}&{}{}&{}{ + \frac{2}{c}\ln k + \frac{2}{c}\ln k - {\sigma _3}{W_3}(t)}\\ {}&{} = &{}{\ln P(0) + \left[ {\frac{{\theta _1^2}}{{2(1 - c)\sigma _1^2}} + \frac{{\theta _2^2}}{{2(1 - c)\sigma _2^2}} - ({\lambda _1} + {\gamma _1} + \mu + \frac{1}{2}\sigma _3^2)} \right] t}\\ {}&{}{}&{}{ + \frac{2}{c}\ln k + \frac{2}{c}\ln k - {\sigma _3}{W_3}(t).} \end{array}\ \end{aligned} \end{aligned}$$

Hence, for $k-1 \le t \le k$, $\frac{\ln P(t)}{t}$ can be obtained as:

20 $$\begin{aligned} \begin{aligned} \ \begin{array}{*{20}{l}} {\frac{{\ln P(t)}}{t}}&{} \le &{}{\frac{{\ln P(0)}}{t} + \frac{{\theta _1^2}}{{2(1 - c)\sigma _1^2}} + \frac{{\theta _2^2}}{{2(1 - c)\sigma _2^2}} - ({\lambda _1} + {\gamma _1} + \mu + \frac{1}{2}\sigma _3^2)}\\ {}&{}{}&{}{ + \frac{2}{c} \cdot \frac{{\ln k}}{{k - 1}} + \frac{2}{c} \cdot \frac{{\ln k}}{{k - 1}} - {\sigma _3}\frac{{{W_3}(t)}}{t}.} \end{array}\ \end{aligned} \end{aligned}$$

By the strong law of large numbers to the Brownian motion, let $k \rightarrow \infty$ and then $t \rightarrow \infty$, it can be known that $\mathop {\lim }\limits _{t \rightarrow \infty } \sup \frac{{{W_3}(t)}}{t} = 0$.

Therefore

21 $$\begin{aligned} \begin{aligned} \ \mathop {\lim }\limits _{t \rightarrow \infty } \sup \frac{{\ln P(t)}}{t} \le \frac{{\theta _1^2}}{{2(1 - c)\sigma _1^2}} + \frac{{\theta _2^2}}{{2(1 - c)\sigma _2^2}} - ({\lambda _1} + {\gamma _1} + \mu + \frac{1}{2}\sigma _3^2).\ \end{aligned} \end{aligned}$$

Finally, let $c \rightarrow 0$, $\mathop {\lim }\limits _{t \rightarrow \infty } \sup \frac{{\ln {P}(t)}}{t}$ can be obtained as:

22 $$\begin{aligned} \begin{aligned} \ \mathop {\lim }\limits _{t \rightarrow \infty } \sup \frac{{\ln P(t)}}{t} \le \frac{{\theta _1^2}}{{2\sigma _1^2}} + \frac{{\theta _2^2}}{{2\sigma _2^2}} - ({\lambda _1} + {\gamma _1} + \mu + \frac{1}{2}\sigma _3^2).\ \end{aligned} \end{aligned}$$

$$\square $$

### Theorem 3

For any given initial value $({S}(0),{P}(0),{A}(0),{G_1}(0),{G_2}(0)) \in {\mathbb {R}}_ + ^5$, $\mathop {\lim }\limits _{t \rightarrow \infty } \sup \frac{{\ln A(t)}}{t} \le K \left( \sigma _2^2,\sigma _4^2 \right)$ holds a.s.. Further, $K(\sigma _2^2,\sigma _4^2)<0$, then *A*(*t*) tend to 0 exponentially a.s., where $K \left( \sigma _2^2,\sigma _4^2 \right) = \frac{{\theta _2^2}}{{2\sigma _2^2}} - \left( {\lambda _2} + {\gamma _2} + \mu + \frac{1}{2}\sigma _4^2 \right)$.

### Proof

Use $It{\hat{o}}'s$ formula to calculate the differentiation of *A*(*t*) in stochastic system (2), and $d\ln A(t)$ can be written as:

23 $$\begin{aligned} \begin{aligned} \ d\ln A(t) = \left[ {{\alpha _2}S - \beta {\theta _2}P - ({\lambda _2} + {\gamma _2} + \mu ) - \frac{1}{2}{\beta ^2}\sigma _2^2{P^2} - \frac{1}{2}\sigma _4^2} \right] dt - \beta {\sigma _2}Pd{W_2} - {\sigma _4}d{W_4}.\ \end{aligned} \end{aligned}$$

Thus, $\ln {A}(t)$ can be denoted as:

24 $$\begin{aligned} \begin{aligned} \ \begin{array}{*{20}{l}} {\ln A(t)}&{} = &{}{\ln A(0) + \int _0^t {\left[ {{\alpha _2}S(u) - \beta {\theta _2}P(u) - ({\lambda _2} + {\gamma _2} + \mu ) - \frac{1}{2}{\beta ^2}\sigma _2^2{P^2}(u) - \frac{1}{2}\sigma _4^2} \right] du} }\\ {}&{}{}&{}{ - \int _0^t {\beta {\sigma _2}P(u)d{W_2}(u)} - {\sigma _4}d{W_4}(t).} \end{array}\ \end{aligned} \end{aligned}$$

Denote

25 $$\begin{aligned} \begin{aligned} \ {\Phi _3}(t) = \int _0^t {\beta {\sigma _2}P(u)d{W_2}(u)},\ \end{aligned} \end{aligned}$$

${\Phi _3}(t)$ is continuous local martingale. The quadratic variation of ${\Phi _3}(t)$ can be denoted as:

26 $$\begin{aligned} \begin{aligned} \ \left\langle {{\Phi _3}(t)} \right\rangle = \sigma _2^2\int _0^t {{\beta ^2}{P^2}(u)du}.\ \end{aligned} \end{aligned}$$

Similar to Theorem 2, for all $t \in [0,k]$, one can obtain

27 $$\begin{aligned} \begin{aligned} \ \int _0^t {\beta {\sigma _2}P(u)d{W_2}(u) \le \frac{1}{2}} c\sigma _2^2\int _0^t {{\beta ^2}{P^2}(u)du} + \frac{2}{c}\ln k.\ \end{aligned} \end{aligned}$$

And then, it can be obtained that

28 $$\begin{aligned} \begin{aligned} \ \ln A(t) \le \ln A(0) + \int _0^t {\left[ \begin{array}{l} {\alpha _2}S(u) - \beta {\theta _2}P(u) - ({\lambda _2} + {\gamma _2} + \mu )\\ - \frac{1}{2}(1 - c){\beta ^2}\sigma _2^2{P^2}(u) - \frac{1}{2}\sigma _4^2 \end{array} \right] du + \frac{2}{c}\ln k - {\sigma _4}{W_4}(t),} \ \end{aligned} \end{aligned}$$

noting that

29 $$\begin{aligned} \begin{aligned} \ - \beta {\theta _2}P(u) - \frac{1}{2}(1 - c){\beta ^2}\sigma _2^2{P^2}(u) \le \frac{{\theta _2^2}}{{2(1 - c)\sigma _2^2}}.\ \end{aligned} \end{aligned}$$

Substituting Eq. (29) into Eq. (28), $\ln A(t)$ can be written as:

30 $$\begin{aligned} \begin{aligned} \ \begin{array}{*{20}{l}} {\ln A(t)}&{} \le &{}{\ln A(0) + \int _0^t {\left[ {\frac{{\theta _2^2}}{{2(1 - c)\sigma _2^2}} - ({\lambda _2} + {\gamma _2} + \mu + \frac{1}{2}\sigma _4^2)} \right] du + \frac{2}{c}\ln k - {\sigma _4}{W_4}(t)} }\\ {}&{} = &{}{\ln A(0) + \left[ {\frac{{\theta _2^2}}{{2(1 - c)\sigma _2^2}} - ({\lambda _2} + {\gamma _2} + \mu + \frac{1}{2}\sigma _4^2)} \right] t + \frac{2}{c}\ln k - {\sigma _4}{W_4}(t).} \end{array}\ \end{aligned} \end{aligned}$$

Hence, for $k-1 \le t \le k$, $\frac{\ln A(t)}{t}$ can be obtained as:

31 $$\begin{aligned} \begin{aligned} \ \frac{{\ln A(t)}}{t} \le \frac{{\ln A(0)}}{t} + \frac{{\theta _2^2}}{{2(1 - c)\sigma _2^2}} - \left( {\lambda _2} + {\gamma _2} + \mu + \frac{1}{2}\sigma _4^2 \right) + \frac{2}{c} \cdot \frac{{\ln k}}{{k - 1}} - {\sigma _4}\frac{{{W_4}(t)}}{t}.\ \end{aligned} \end{aligned}$$

By the strong law of large numbers to the Brownian motion, let $k \rightarrow \infty$ and then $t \rightarrow \infty$, it can be known that

32 $$\begin{aligned} \begin{aligned} \ \mathop {\lim }\limits _{t \rightarrow \infty } \sup \frac{{\ln A(t)}}{t} \le \frac{{\theta _2^2}}{{2(1 - c)\sigma _2^2}} - ({\lambda _2} + {\gamma _2} + \mu + \frac{1}{2}\sigma _4^2).\ \end{aligned} \end{aligned}$$

Finally, let $c \rightarrow 0$, $\mathop {\lim }\limits _{t \rightarrow \infty } \sup \frac{{\ln {A}(t)}}{t}$ can be obtained as:

33 $$\begin{aligned} \begin{aligned} \ \mathop {\lim }\limits _{t \rightarrow \infty } \sup \frac{{\ln A(t)}}{t} \le \frac{{\theta _2^2}}{{2\sigma _2^2}} - \left( {\lambda _2} + {\gamma _2} + \mu + \frac{1}{2}\sigma _4^2 \right) .\ \end{aligned} \end{aligned}$$

$$\square $$

### Remark 1

$K \left( \sigma _1^2,\sigma _2^2,\sigma _3^2 \right) = \frac{{\theta _1^2}}{{2\sigma _1^2}} + \frac{{\theta _2^2}}{{2\sigma _2^2}} - \left( {\lambda _1} + {\gamma _1} + \mu + \frac{1}{2}\sigma _3^2 \right)$ and $K(\sigma _2^2,\sigma _4^2) = \frac{{\theta _2^2}}{{2\sigma _2^2}} - \left( {\lambda _2} + {\gamma _2} + \mu + \frac{1}{2}\sigma _4^2 \right)$ are decreasing in ${\sigma _1^2}$, ${\sigma _2^2}$, ${\sigma _3^2}$ and ${\sigma _4^2}$. The investor sentiment will disappearance eventually if ${\sigma _1^2}$, ${\sigma _2^2}$, ${\sigma _3^2}$ and ${\sigma _4^2}$ are large enough, where $K \left( \sigma _1^2,\sigma _2^2,\sigma _3^2 \right) <0$ and $K \left( \sigma _2^2,\sigma _4^2 \right) <0$.

## A sufficient condition for the stationary distribution

Theorem 4 gives the unique stationary distribution of the existence of stochastic system (2). This also means the stability in a stochastic sense.

### Theorem 4

If the stochastic system (2) with initial condition $({S}(0),{P}(0),{A}(0),{G_1}(0),{G_2}(0)) \in {\mathbb {R}}_ + ^5$ and the following conditions are satisfied

34 $$\begin{aligned} \begin{aligned} \ 0< \Gamma < \min \left( {\xi _1}{S^2},{\xi _2}{P^2},{\xi _3}{A^2},{\xi _4}G_1^2,{\xi _5}G_2^2 \right) ,\ \end{aligned} \end{aligned}$$

where

35 $$\begin{aligned} \begin{aligned} \ \begin{array}{*{20}{l}} \Gamma &{} = &{}{\frac{1}{2}\sigma _3^2{P^*} + \frac{1}{2}\sigma _4^2{A^*},}\\ {{\xi _1}}&{} = &{}{\mu - \alpha _1^2\sigma _1^2,}\\ {{\xi _2}}&{} = &{}{({\lambda _1} + {\gamma _1} + \mu ) - \left( {\beta ^2}\sigma _2^2 + \sigma _3^2 \right) ,}\\ {{\xi _3}}&{} = &{}{({\lambda _2} + {\gamma _2} + \mu ) - \left( {\beta ^2}\sigma _2^2 + \sigma _4^2 \right) ,}\\ {{\xi _4}}&{} = &{}{{\varepsilon _1} + \mu ,}\\ {{\xi _5}}&{} = &{}{{\varepsilon _2} + \mu .} \end{array}\ \end{aligned} \end{aligned}$$

then the stationary distribution $\pi$ exists, and the solution of stochastic system (2) is ergodic.

By the investor sentiment-existence equilibrium point $E^* = ({S^*},{P^*},{A^*},{G_1^*},{G_2^*})$ can be get that

36 $$\begin{aligned} \begin{aligned} \ \mathop {\lim }\limits _{t \rightarrow \infty } \frac{1}{t}E\int _0^t {\left[ \begin{array}{l} {\xi _1}{(S(u) - {S^*})^2} + {\xi _2}{(P(u) - {P^*})^2} + {\xi _3}{(A(u) - {A^*})^2}\\ + {\xi _4}{({G_1}(u) - G_1^*)^2} + {\xi _5}{({G_2}(u) - G_2^*)^2} \end{array} \right] } du < \Gamma .\ \end{aligned} \end{aligned}$$

### Proof

Define a $${\mathcal {C}}^2$$ function *V*:

37 $$\begin{aligned} \begin{aligned} \ \Theta (S,P,A,{G_1},{G_2}) = {\Theta _1}(P) + {\Theta _2}(A) + {\Theta _3}({G_1}) + {\Theta _4}({G_2}) + {\Theta _5}(S,P,A,{G_1},{G_2}),\ \end{aligned} \end{aligned}$$

where

38 $$\begin{aligned} \begin{aligned} \ \begin{array}{l} {\Theta _1}(P) = P - {P^*} - {P^*}\ln \frac{P}{{{P^*}}},\\ {\Theta _2}(A) = A - {A^*} - {A^*}\ln \frac{A}{{{A^*}}},\\ {\Theta _3}({G_1}) = {G_1} - G_1^* - G_1^*\ln \frac{{{G_1}}}{{G_1^*}},\\ {\Theta _4}({G_2}) = {G_2} - G_2^* - G_2^*\ln \frac{{{G_2}}}{{G_2^*}},\\ {\Theta _5}(S,P,A,{G_1},{G_2}) = \frac{1}{2}{(S + P + A + {G_1} + {G_2} - {S^*} - {P^*} - {A^*} - G_1^* - G_2^*)^2}. \end{array}\ \end{aligned} \end{aligned}$$

The differential *L* operator to $\Theta _1$ can be calculated as:

39 $$\begin{aligned} \begin{aligned} \ \begin{array}{*{20}{l}} {L{\Theta _1}}&{} = &{}{\left[ {{\alpha _1}{\theta _1}SP + \beta {\theta _2}AP - ({\lambda _1} + {\gamma _1} + \mu )P} \right] \frac{{\partial {\Theta _1}}}{{\partial P}} + \frac{1}{2} \left( \alpha _1^2\sigma _1^2{S^2}{P^2} + {\beta ^2}\sigma _2^2{A^2}{P^2} + \sigma _3^2{P^2}\right) \frac{{{\partial ^2}{\Theta _1}}}{{\partial {P^2}}}}\\ {}&{} = &{}{(P - {P^*})\left[ {{\alpha _1}{\theta _1}S + \beta {\theta _2}A - ({\lambda _1} + {\gamma _1} + \mu )} \right] + \frac{1}{2}\alpha _1^2\sigma _1^2{S^2}{P^*} + \frac{1}{2}{\beta ^2}\sigma _2^2{A^2}{P^*} + \frac{1}{2}\sigma _3^2{P^*},} \end{array}\ \end{aligned} \end{aligned}$$

According to $E^* = ({S^*},{P^*},{A^*},{G_1^*},{G_2^*})$, it is easy to get that

40 $$\begin{aligned} \begin{aligned} \ {\lambda _1} + {\gamma _1} + \mu = {\alpha _1}{\theta _1}{S^*} + \beta {\theta _2}{A^*},\ \end{aligned} \end{aligned}$$

and then, ${L{\Theta _1}}$ can be expressed as:

41 $$\begin{aligned} \begin{aligned} L{\Theta _1} = (P - {P^*})\left[ {{\alpha _1}{\theta _1}(S - {S^*}) + \beta {\theta _2}(A - {A^*})} \right] + \frac{1}{2}\alpha _1^2\sigma _1^2{S^2}{P^*} + \frac{1}{2}{\beta ^2}\sigma _2^2{A^2}{P^*} + \frac{1}{2}\sigma _3^2{P^*}, \end{aligned} \end{aligned}$$

where $${\alpha _1}{\theta _1}(S - {S^*}) \ge 0$$ and $$\beta {\theta _2}(A - {A^*}) \ge 0$$.

By simple calculation, one can get

42 $$\begin{aligned} \begin{aligned} \ \begin{array}{*{20}{l}} {L{\Theta _1}}&{} \le &{}{{\alpha _1}{\theta _1}(S - {S^*})(P - {P^*}) + \beta {\theta _2}(A - {A^*})(P - {P^*}) + \frac{1}{2}\alpha _1^2\sigma _1^2{{\left[ {(S - {S^*}) + {S^*}} \right] }^2}{P^*}}\\ {}&{}{}&{}{ + \frac{1}{2}{\beta ^2}\sigma _2^2{{\left[ {(A - {A^*}) + {A^*}} \right] }^2}{P^*} + \frac{1}{2}\sigma _3^2{P^*},} \end{array}\ \end{aligned} \end{aligned}$$

due to $\frac{1}{2}{(x + y)^2} \le {x^2} + {y^2}$, it is easy to obtain that

43 $$\begin{aligned} \begin{aligned} \begin{array}{*{20}{l}} {L{\Theta _1}}&{} \le &{}{{\alpha _1}{\theta _1}(S - {S^*})(P - {P^*}) + \beta {\theta _2}(A - {A^*})(P - {P^*}) + \alpha _1^2\sigma _1^2{{(S - {S^*})}^2}{P^*}}\\ {}&{}{}&{}{ + {\beta ^2}\sigma _2^2{{(A - {A^*})}^2}{P^*} + \frac{1}{2}\sigma _3^2{P^*}.} \end{array}\ \end{aligned} \end{aligned}$$

Similarly, ${L{\Theta _2}}$ can be obtained that

44 $$\begin{aligned} \begin{aligned} \ L{\Theta _2} \le {\alpha _2}(S - {S^*})(A - {A^*}) - \beta {\theta _2}(A - {A^*})(P - {P^*}) + {\beta ^2}\sigma _2^2{(P - {P^*})^2}{A^*} + \frac{1}{2}\sigma _4^2{A^*}.\ \end{aligned} \end{aligned}$$

Next, the differential *L* operator to $\Theta _3$ can be calculated as:

45 $$\begin{aligned} \begin{aligned} \ \begin{array}{*{20}{l}} {L{\Theta _3}}&{} = &{}{({\lambda _1}P - {\varepsilon _1}{G_1} - \mu {G_1})\frac{{\partial {\Theta _3}}}{{\partial {G_1}}} + \frac{1}{2}\sigma _3^2{P^2}\frac{{{\partial ^2}{\Theta _3}}}{{\partial G_1^2}}}\\ {}&{} = &{}{({G_1} - G_1^*)(\frac{{{\lambda _1}P}}{{{G_1}}} - {\varepsilon _1} - \mu ) + \frac{1}{2}\sigma _3^2{P^2}.} \end{array}\ \end{aligned} \end{aligned}$$

According to $E^* = ({S^*},{P^*},{A^*},{G_1^*},{G_2^*})$, it is easy to get that

46 $$\begin{aligned} \begin{aligned} \ {\varepsilon _1} + \mu = \frac{{{\lambda _1}{P^*}}}{{G_1^*}},\ \end{aligned} \end{aligned}$$

and $L{\Theta _3}$ can be obtained as:

47 $$\begin{aligned} \begin{aligned} \ \begin{array}{*{20}{l}} {L{\Theta _3}}&{} = &{}{({G_1} - G_1^*)(\frac{{{\lambda _1}P}}{{{G_1}}} - \frac{{{\lambda _1}{P^*}}}{{G_1^*}}) + \frac{1}{2}\sigma _3^2{P^2}}\\ {}&{} = &{}{({G_1} - G_1^*)\left[ { - \frac{{{\lambda _1}P({G_1} - G_1^*)}}{{{G_1}G_1^*}} + \frac{{{\lambda _1}(P - {P^*})}}{{G_1^*}}} \right] + \frac{1}{2}\sigma _3^2{P^2}.} \end{array}\ \end{aligned} \end{aligned}$$

where $$\frac{{{\lambda _1}P({G_1} - G_1^*)}}{{{G_1}G_1^*}} \ge 0$$ and $${G_1}^*>0$$.

By simple calculation, one can get

48 $$\begin{aligned} \begin{aligned} \ L{\Theta _3} \le {\lambda _1}(P - {P^*})({G_1} - G_1^*) + \frac{1}{2}\sigma _3^2{\left[ {(P - {P^*}) + {P^*}} \right] ^2},\ \end{aligned} \end{aligned}$$

due to $\frac{1}{2}{(x + y)^2} \le {x^2} + {y^2}$, it is easy to obtain that

49 $$\begin{aligned} \begin{aligned} \ L{\Theta _3} \le {\lambda _1}(P - {P^*})({G_1} - G_1^*) + \sigma _3^2{(P - {P^*})^2}.\ \end{aligned} \end{aligned}$$

Similarly, ${L{\Theta _4}}$ can be obtained that

50 $$\begin{aligned} \begin{aligned} \ L{\Theta _4} \le {\lambda _2}(A - {A^*})({G_2} - G_2^*) + \sigma _4^2{(A - {A^*})^2}.\ \end{aligned} \end{aligned}$$

Finally, the differential *L* operator to $\Theta _5$ can be calculated as:

51 $$\begin{aligned} \begin{aligned} \ \begin{array}{*{20}{l}} {L{\Theta _5}}&{} = &{}{\left( \begin{array}{l} S + P + A + {G_1} + {G_2} - {S^*}\\ - {P^*} - {A^*} - G_1^* - G_2^* \end{array} \right) \left[ \begin{array}{l} B - \mu S - ({\lambda _1} + {\gamma _1} + \mu )P - ({\lambda _2} + {\gamma _2} + \mu )A\\ - ({\varepsilon _1} + \mu ){G_1} - ({\varepsilon _2} + \mu ){G_2} \end{array} \right] }\\ {}&{} = &{}{\left( \begin{array}{l} S - {S^*} + P - {P^*} + A - {A^*}\\ + {G_1} - G_1^* + {G_2} - G_2^* \end{array} \right) \left[ \begin{array}{l} - \mu (S - {S^*}) - ({\lambda _1} + {\gamma _1} + \mu )(P - {P^*})\\ - ({\lambda _2} + {\gamma _2} + \mu )(A - {A^*}) - ({\varepsilon _1} + \mu )({G_1} - G_1^*)\\ - ({\varepsilon _2} + \mu )({G_2} - G_2^*) \end{array} \right] }\\ {}&{} \le &{}{ - \mu {{(S - {S^*})}^2} - ({\lambda _1} + {\gamma _1} + \mu )(S - {S^*})(P - {P^*}) - ({\lambda _2} + {\gamma _2} + \mu )(S - {S^*})(A - {A^*})}\\ {}&{}{}&{}{ - ({\varepsilon _1} + \mu )(S - {S^*})({G_1} - G_1^*) - ({\varepsilon _2} + \mu )(S - {S^*})({G_2} - G_2^*) - \mu (S - {S^*})(P - {P^*})}\\ {}&{}{}&{}{ - ({\lambda _1} + {\gamma _1} + \mu ){{(P - {P^*})}^2} - ({\lambda _2} + {\gamma _2} + \mu )(A - {A^*})(P - {P^*}) - \mu (S - {S^*})({G_2} - G_2^*)}\\ {}&{}{}&{}{ - ({\varepsilon _2} + \mu )(P - {P^*})({G_2} - G_2^*) - \mu (S - {S^*})(A - {A^*}) - ({\lambda _1} + {\gamma _1} + \mu )(P - {P^*})(A - {A^*})}\\ {}&{}{}&{}{ - ({\lambda _2} + {\gamma _2} + \mu ){{(A - {A^*})}^2} - ({\varepsilon _1} + \mu )(A - {A^*})({G_1} - G_1^*) - ({\varepsilon _2} + \mu )(A - {A^*})({G_2} - G_2^*)}\\ {}&{}{}&{}{ - \mu (S - {S^*})({G_1} - G_1^*) - ({\lambda _1} + {\gamma _1} + \mu )(P - {P^*})({G_1} - G_1^*) - ({\varepsilon _1} + \mu )(P - {P^*})({G_1} - G_1^*)}\\ {}&{}{}&{}{ - ({\varepsilon _1} + \mu ){{({G_1} - G_1^*)}^2} - ({\varepsilon _2} + \mu )({G_1} - G_1^*)({G_2} - G_2^*) - ({\lambda _2} + {\gamma _2} + \mu )(A - {A^*})({G_1} - G_1^*)}\\ {}&{}{}&{}{ - ({\lambda _1} + {\gamma _1} + \mu )(P - {P^*})({G_2} - G_2^*) - ({\lambda _2} + {\gamma _2} + \mu )(A - {A^*})({G_2} - G_2^*) - ({\varepsilon _2} + \mu ){{({G_2} - G_2^*)}^2}}\\ {}&{}{}&{}{ - ({\varepsilon _1} + \mu )({G_1} - G_1^*)({G_2} - G_2^*).} \end{array}\ \end{aligned} \end{aligned}$$

Substitute Eqs. (43), (44), (49), (50) and (51) into Eq. (37) to get

52 $$\begin{aligned} \begin{aligned} \ \begin{array}{*{20}{l}} {\Theta (S,P,A,{G_1},{G_2})}&{} \le &{}{\alpha _1^2\sigma _1^2{{(S - {S^*})}^2} + {\beta ^2}\sigma _2^2{{(A - {A^*})}^2} + \frac{1}{2}\sigma _3^2{P^*} + {\beta ^2}\sigma _2^2{{(P - {P^*})}^2}}\\ {}&{}{}&{}{ + \frac{1}{2}\sigma _4^2{A^*} + \sigma _3^2{{(P - {P^*})}^2} + \sigma _4^2{{(A - {A^*})}^2} - \mu {{(S - {S^*})}^2}}\\ {}&{}{}&{}{ - ({\lambda _1} + {\gamma _1} + \mu ){{(P - {P^*})}^2} - ({\lambda _2} + {\gamma _2} + \mu ){{(A - {A^*})}^2}}\\ {}&{}{}&{}{ - ({\varepsilon _1} + \mu ){{({G_1} - G_1^*)}^2} - ({\varepsilon _2} + \mu ){{({G_2} - G_2^*)}^2}}\\ {}&{} = &{}{(\alpha _1^2\sigma _1^2 - \mu ){{(S - {S^*})}^2} + \left[ {{\beta ^2}\sigma _2^2 + \sigma _3^2 - ({\lambda _1} + {\gamma _1} + \mu )} \right] {{(P - {P^*})}^2}}\\ {}&{}{}&{}{ + \left[ {{\beta ^2}\sigma _2^2 + \sigma _4^2 - ({\lambda _2} + {\gamma _2} + \mu )} \right] {{(A - {A^*})}^2} - ({\varepsilon _1} + \mu ){{({G_1} - G_1^*)}^2}}\\ {}&{}{}&{}{ - ({\varepsilon _2} + \mu ){{({G_2} - G_2^*)}^2} + \frac{1}{2}\sigma _3^2{P^*} + \frac{1}{2}\sigma _4^2{A^*}.} \end{array}\ \end{aligned} \end{aligned}$$

By Eq. (34), the ellipsoid

53 $$\begin{aligned} \begin{aligned} \ - {\xi _1}{(S - {S^*})^2} - {\xi _2}{(P - {P^*})^2} - {\xi _3}{(A - {A^*})^2} - {\xi _4}{({G_1} - G_1^*)^2} - {\xi _5}{({G_2} - G_2^*)^2} + \Gamma = 0\ \end{aligned} \end{aligned}$$

lies entirely in ${\mathbb {R}}_ + ^5$. According to^37^, it is easy to know that stochastic system (2) has a stable stationary distribution. $\square$

### Remark 2

By Theorem 4, there exist

54 $$\begin{aligned} \begin{aligned} \ \begin{array}{*{20}{l}} {\mathop {\lim }\limits _{({\sigma _1},{\sigma _2},{\sigma _3},{\sigma _4}) \rightarrow 0} \Gamma }&{} = &{}{0,}\\ {\mathop {\lim }\limits _{({\sigma _1},{\sigma _2},{\sigma _3},{\sigma _4}) \rightarrow 0} {\xi _1}}&{} = &{}{\mu> 0,}\\ {\mathop {\lim }\limits _{({\sigma _1},{\sigma _2},{\sigma _3},{\sigma _4}) \rightarrow 0} {\xi _2}}&{} = &{}{{\lambda _1} + {\gamma _1} + \mu> 0,}\\ {\mathop {\lim }\limits _{({\sigma _1},{\sigma _2},{\sigma _3},{\sigma _4}) \rightarrow 0} {\xi _3}}&{} = &{}{{\lambda _2} + {\gamma _2} + \mu> 0,}\\ {\mathop {\lim }\limits _{({\sigma _1},{\sigma _2},{\sigma _3},{\sigma _4}) \rightarrow 0} {\xi _4}}&{} = &{}{{\varepsilon _1} + \mu> 0,}\\ {\mathop {\lim }\limits _{({\sigma _1},{\sigma _2},{\sigma _3},{\sigma _4}) \rightarrow 0} {\xi _5}}&{} = &{}{{\varepsilon _2} + \mu > 0,} \end{array}\ \end{aligned} \end{aligned}$$

so that the solution of stochastic system (2) fluctuates around $E^*$. Moreover, the difference between deterministic system and stochastic system (2) decreases with the values of ${\sigma _1}$, ${\sigma _2}$, ${\sigma _3}$ and ${\sigma _4}$ decreasing.

## The stochastic optimal control model

Based on the random investor sentiment contagion model established above, the paper recognizes that positive investor sentiment significantly promotes economic and social development. Conversely, when managers need to regulate investor sentiment, effective measures of regulatory isolation can be implemented. In this view, the paper introduces two control objectives aimed at facilitating the transformation of positive investor sentiment disseminators and groups under regulatory isolation. Consequently, the four constants of proportionality in the model $\theta _1, \theta _2, \lambda _1$ and $\lambda _2$ were changed into control variables $\theta _1(t), \theta _2(t), \lambda _1(t)$ and $\lambda _2(t)$.

Hence, the objective function can be proposed as:

55 $$\begin{aligned} \begin{aligned} \ J(P,{G_1},{G_2}) = \int _0^{{t_f}} {\left[ P(t) + {G_1}(t) + {G_2}(t) - {{{{c_1}}}/{2}}\theta _1^2(t) - {{{{c_2}}}/{2}}\theta _2^2(t) - {{{{c_3}}}/{2}}\lambda _1^2(t) - {{{{c_4}}}/{2}}\lambda _2^2(t) \right] ,} \ \end{aligned} \end{aligned}$$

and the objective function satisfy the state system as:

56 $$\begin{aligned} \begin{aligned} \ \left\{ {\begin{array}{*{20}{l}} {dS(t)}&{} = &{}{\left[ {B - {\alpha _1}{\theta _1}(t)SP - {\alpha _2}SA - \mu S} \right] dt - {\alpha _1}{\sigma _1}SPd{W_1}(t),}\\ {dP(t)}&{} = &{}\begin{array}{l} \left[ {{\alpha _1}{\theta _1}(t)SP + \beta {\theta _2}(t)AP - {\lambda _1}(t)P - {\gamma _1}P - \mu P} \right] dt\\ + {\alpha _1}{\sigma _1}SPd{W_1}(t) + \beta {\sigma _2}APd{W_2}(t) - {\sigma _3}Pd{W_3}(t), \end{array}\\ {dA(t)}&{} = &{}\begin{array}{l} \left[ {{\alpha _2}SA - \beta {\theta _2}(t)AP - {\lambda _2}(t)A - {\gamma _2}A - \mu A} \right] dt\\ - \beta {\sigma _2}APd{W_2}(t) - {\sigma _4}Ad{W_4}(t), \end{array}\\ {d{G_1}(t)}&{} = &{}{\left[ {{\lambda _1}(t)P - {\varepsilon _1}{G_1} - \mu {G_1}} \right] dt + {\sigma _3}Pd{W_3}(t),}\\ {d{G_2}(t)}&{} = &{}{\left[ {{\lambda _2}(t)A - {\varepsilon _2}{G_2} - \mu {G_2}} \right] dt + {\sigma _4}Ad{W_4}(t).} \end{array}} \right. \ \end{aligned} \end{aligned}$$

The initial conditions for system (56) are satisfied:

57 $$\begin{aligned} \begin{aligned} \ S(0) = {S_0},P(0) = {P_0},A(0) = {A_0},{G_1}(0) = {G_{1,0}},{G_2}(0) = {G_{2,0}},\ \end{aligned} \end{aligned}$$

where

58 $$\begin{aligned} \begin{aligned} \ \begin{array}{*{20}{l}} {{\theta _1}(t),{\theta _2}(t),{\lambda _1}(t),{\lambda _2}(t)}&{} \in &{}{U \buildrel \Delta \over = \left\{ \begin{array}{l} ({\theta _1},{\theta _2},{\lambda _1},{\lambda _2})|({\theta _1}(t),{\theta _2}(t),{\lambda _1}(t),{\lambda _2}(t))\\ measurable,0 \le {\theta _1}(t),{\theta _2}(t),{\lambda _1}(t),{\lambda _2}(t) \le 1,\forall t \in [0,{t_f}] \end{array} \right\} ,} \end{array}\ \end{aligned} \end{aligned}$$

while *U* is the admissible control set. 0 and $t_f$ are the time interval. The control strength and importance of control measures are expressed as $c_1$, $c_2$, $c_3$ and $c_4$, which are the positive weight coefficients.

### Theorem 5

There exists an optimal control pair $\left( {\theta _1^*,\theta _2^*,\lambda _1^*,\lambda _2^*} \right) \in U$, so that the function is established as:

59 $$\begin{aligned} \begin{aligned} \ J(\theta _1^*,\theta _2^*,\lambda _1^*,\lambda _2^*) = \max \{ J({\theta _1},{\theta _2},{\lambda _1},{\lambda _2}):({\theta _1},{\theta _2},{\lambda _1},{\lambda _2}) \in U\}.\ \end{aligned} \end{aligned}$$

### Proof

Let $$X(t) = {(S(t),P(t),A(t),{G_1}(t),{G_2}(t),{R_1}(t),{R_2}(t))^T}$$ and

60 $$\begin{aligned} \begin{aligned} \ \begin{array}{*{20}{l}} {L\left( {t;X(t),{\theta _1}(t),{\theta _2}(t),{\lambda _1}(t),{\lambda _2}(t)} \right) }&{} = &{}{P(t) + {G_1}(t) + {G_2}(t) - {{{c_1}}/{2\theta _1^2(t)}}}\\ {}&{}\quad - &{}{{{{c_2}}/{2\theta _2^2(t) - {{{c_3}}/{2\lambda _1^2(t) - {{{c_4}}/{2\lambda _2^2(t).}}}}}}} \end{array}\ \end{aligned} \end{aligned}$$

The following five conditions must be satisfied and then the optimal control pair is existence.

(i) The set of control variables and state variables is nonempty.
(ii) The control set *U* is convex and closed.
(iii) The right-hand side of the state system is bounded by a linear function in the state and control variables.
(iv) The integrand of the objective functional is convex on *U*.
(v) There exist constants $d_1,d_2>0$ and $\rho >1$ such that the integrand of the objective functional satisfied:
  61 $$\begin{aligned} \begin{aligned} \ - L(t;X(t),{\theta _1};{\theta _2};{\lambda _1};{\lambda _2}) \ge {d_1}{({\left| {{\theta _1}} \right| ^2} + {\left| {{\theta _2}} \right| ^2} + {\left| {{\lambda _1}} \right| ^2} + {\left| {{\lambda _2}} \right| ^2})^{{{\rho } / {2}}}} - {d_2}. \end{aligned} \end{aligned}$$

It is clearly that conditions (i)–(iii) established. Then, the condition (iv) can be easily established such that

62 $$\begin{aligned} \begin{aligned} \ \begin{array}{*{20}{l}} {S' \le B,P' \le {\alpha _1}{\theta _1}(t)SP + \beta {\theta _2}(t)AP,A' \le {\alpha _2}SA,{{G'}_1} \le {\lambda _1}(t)P,{{G'}_2} \le {\lambda _2}(t)A.} \end{array}\ \end{aligned} \end{aligned}$$

Next, for any $t \ge 0$, there is a positive constant *M* which is satisfied $|X(t)| \le M$, therefore

63 $$\begin{aligned} \begin{aligned} \ \begin{array}{*{20}{l}} { - L(t;X(t),{\theta _1};{\theta _2};{\lambda _1};{\lambda _2})}&{} = &{}{{{({c_1}\theta _1^2(t) + {c_2}\theta _2^2(t) + {c_3}\lambda _1^2(t) + {c_4}\lambda _2^2(t))} / 2}}\\ {}&{}{}&{}{ - P(t) - {G_1}(t) - {G_2}(t)}\\ {}&{} \ge &{}{{d_1}{{({{\left| {{\theta _1}} \right| }^2} + {{\left| {{\theta _2}} \right| }^2} + {{\left| {{\lambda _1}} \right| }^2} + {{\left| {{\lambda _2}} \right| }^2})}^{{{\rho }/{2}}}} - 2M.} \end{array} \end{aligned} \end{aligned}$$

Let ${d_1} = \min \left\{ {\frac{{{c_1}}}{2},\frac{{{c_2}}}{2},\frac{{{c_3}}}{2},\frac{{{c_4}}}{2}} \right\} ,{d_2} = 2M$ and $\rho =2$, then condition (v) is established. Hence, the optimal control can be realized. $\square$

### Theorem 6

There exist adjoint variables $\delta _1,\delta _2,\delta _3,\delta _4,\delta _5$ for the optimal control pair $\left( {\theta _1^*,\theta _2^*,\lambda _1^*,\lambda _2^*} \right)$ that satisfy:

64 $$\begin{aligned} \begin{aligned} \ \left\{ {\begin{array}{*{20}{l}} {\frac{{d{\delta _1}}}{{dt}}}&{} = &{}{\left[ \begin{array}{l} \left( {{\delta _1} - {\delta _2}} \right) {\alpha _1}{\theta _1}(t)P + \left( {{\delta _1} - {\delta _3}} \right) {\alpha _2}A\\ + {\delta _1}\mu + ({\zeta _1} - {\zeta _2}){\alpha _1}{\sigma _1}P \end{array} \right] dt - {\zeta _1}d{W_1},}\\ {\frac{{d{\delta _2}}}{{dt}}}&{} = &{}{\left[ \begin{array}{l} 1 + \left( {{\delta _1} - {\delta _2}} \right) {\alpha _1}{\theta _1}(t)S + \left( {{\delta _3} - {\delta _2}} \right) \beta {\theta _2}(t)A\\ + \left( {{\delta _2} - {\delta _4}} \right) {\lambda _1}(t) + \left( {{\delta _2} - {\delta _2}} \right) {\gamma _1} + {\delta _2}\mu - {\zeta _4}{\sigma _3}\\ + ({\zeta _1} - {\zeta _2}){\alpha _1}{\sigma _1}S + ({\zeta _3} - {\zeta _2})\beta {\sigma _2}A + {\zeta _2}{\sigma _3} \end{array} \right] dt + {\zeta _2}d{W_1} + {\zeta _2}d{W_2} - {\zeta _2}d{W_3},}\\ {\frac{{d{\delta _3}}}{{dt}}}&{} = &{}{\left[ \begin{array}{l} \left( {{\delta _1} - {\delta _3}} \right) {\alpha _2}S + \left( {{\delta _3} - {\delta _2}} \right) \beta {\theta _2}(t)P\\ + \left( {{\delta _3} - {\delta _5}} \right) {\lambda _2}(t) + \left( {{\delta _3} - {\delta _5}} \right) {\gamma _2} + {\delta _3}\mu \\ ({\zeta _3} - {\zeta _2})\beta {\sigma _2}P + {\zeta _3}{\sigma _4} - {\zeta _5}{\sigma _4} \end{array} \right] dt - {\zeta _3}d{W_2} - {\zeta _3}d{W_4},}\\ {\frac{{d{\delta _4}}}{{dt}}}&{} = &{}{\left[ {1 + \left( {{\delta _4} - {\delta _6}} \right) {\varepsilon _1} + {\delta _4}\mu } \right] dt + {\zeta _4}d{W_3},}\\ {\frac{{d{\delta _5}}}{{dt}}}&{} = &{}{\left[ {1 + \left( {{\delta _5} - {\delta _7}} \right) {\varepsilon _2} + {\delta _5}\mu } \right] dt + {\zeta _5}d{W_4},} \end{array}} \right. \ \end{aligned} \end{aligned}$$

With boundary conditions:

65 $$\begin{aligned} \begin{aligned} \ {\delta _1}({t_f}) = {\delta _2}({t_f}) = {\delta _3}({t_f}) = {\delta _4}({t_f}) = {\delta _5}({t_f}) = 0.\ \end{aligned} \end{aligned}$$

In addition, the optimal control pair $\left( {\theta _1^*,\theta _2^*,\lambda _1^*,\lambda _2^*} \right)$ of state system (56) can be given by:

66 $$\begin{aligned} \begin{aligned} \ \begin{array}{l} \theta _1^*(t) = \min \left\{ {1,\max \left\{ {0,\frac{{({\delta _1} - {\delta _2}){\alpha _1}SP}}{{{c_1}}}} \right\} } \right\} ,\\ \theta _2^*(t) = \min \left\{ {1,\max \left\{ {0,\frac{{({\delta _3} - {\delta _2})\beta AP}}{{{c_2}}}} \right\} } \right\} ,\\ \lambda _1^*(t) = \min \left\{ {1,\max \left\{ {0,\frac{{({\delta _2} - {\delta _4})P}}{{{c_3}}}} \right\} } \right\} ,\\ \lambda _2^*(t) = \min \left\{ {1,\max \left\{ {0,\frac{{({\delta _3} - {\delta _5})A}}{{{c_4}}}} \right\} } \right\} . \end{array}\ \end{aligned} \end{aligned}$$

### Proof

In order to obtain the expression of optimal control system and optimal control pair, define a Hamiltonian function, which can be written as:

67 $$\begin{aligned} \begin{aligned} \begin{array}{*{20}{l}} H&{} = &{}{ - P(t) - {G_1}(t) - {G_2}(t) + {c_1}/{2}\theta _{1}^{2}(t)(t) + {c_2} /{2}\theta _{2}^{2}(t)(t) + {c_3} / {2}\lambda _{1}^{2}(t) + {c_4}/{2}\lambda _{2}^{2}(t)}\\ {}&{}{}&{}{ + {\delta _1}\left[ {B - {\alpha _1}{\theta _1}(t)SP - {\alpha _2}SA - \mu S} \right] + {\delta _2}\left[ {{\alpha _1}{\theta _1}(t)SP + \beta {\theta _2}(t)AP - {\lambda _1}(t)P - {\gamma _1}P - \mu P} \right] }\\ {}&{}{}&{}{ + {\delta _3}\left[ {{\alpha _2}SA - \beta {\theta _2}(t)AP - {\lambda _2}(t)A - {\gamma _2}A - \mu A} \right] + {\delta _4}\left[ {{\lambda _1}(t)P - {\varepsilon _1}{G_1} - \mu {G_1}} \right] }\\ {}&{}{}&{}{ + {\delta _5}\left[ {{\lambda _2}(t)A - {\varepsilon _2}{G_2} - \mu {G_2}} \right] + ( - {\zeta _1}{\alpha _1}{\sigma _1}SP) + \left[ {{\zeta _2}({\alpha _1}{\sigma _1}SP + \beta {\sigma _2}AP - {\sigma _3}P)} \right] }\\ {}&{}{}&{}{ + \left[ {{\zeta _3}( - \beta {\sigma _2}AP - {\sigma _4}A)} \right] + {\zeta _4}{\sigma _3}P + {\zeta _5}{\sigma _4}A,} \end{array} \end{aligned} \end{aligned}$$

According to the Pontyragin maximum principle, the adjoint system can be written as:

68 $$\begin{aligned} \begin{aligned} \ \frac{{d{\delta _1}}}{{dt}} = - \frac{{\partial H}}{{\partial S}},\frac{{d{\delta _2}}}{{dt}} = - \frac{{\partial H}}{{\partial P}},\frac{{d{\delta _3}}}{{dt}} = - \frac{{\partial H}}{{\partial A}},\frac{{d{\delta _4}}}{{dt}} = - \frac{{\partial H}}{{\partial {G_1}}},\frac{{d{\delta _5}}}{{dt}} = - \frac{{\partial H}}{{\partial {G_2}}},\ \end{aligned} \end{aligned}$$

and the boundary conditions of adjoint system are

69 $$\begin{aligned} \begin{aligned} \ {\delta _1}({t_f}) = {\delta _2}({t_f}) = {\delta _3}({t_f}) = {\delta _4}({t_f}) = {\delta _5}({t_f}) = 0.\ \end{aligned} \end{aligned}$$

Then, the optimal control pair $\left( {\theta _1^*,\theta _2^*,\lambda _1^*,\lambda _2^*} \right)$ can be calculated as:

70 $$\begin{aligned} \begin{aligned} \ \begin{array}{l} \theta _1^*(t) = \min \left\{ {1,\max \left\{ {0,\frac{{({\delta _1} - {\delta _2}){\alpha _1}SP}}{{{c_1}}}} \right\} } \right\} ,\\ \theta _2^*(t) = \min \left\{ {1,\max \left\{ {0,\frac{{({\delta _3} - {\delta _2})\beta AP}}{{{c_2}}}} \right\} } \right\} ,\\ \lambda _1^*(t) = \min \left\{ {1,\max \left\{ {0,\frac{{({\delta _2} - {\delta _4})P}}{{{c_3}}}} \right\} } \right\} ,\\ \lambda _2^*(t) = \min \left\{ {1,\max \left\{ {0,\frac{{({\delta _3} - {\delta _5})A}}{{{c_4}}}} \right\} } \right\} . \end{array}\ \end{aligned} \end{aligned}$$

$$\square $$

### Remark 3

So far, the optimal control system can be got includes state system (56) with the initial conditions *S*(0), *P*(0), *A*(0), 

${{G_1}(0),{G_2}(0)}$ and the adjoint system (64) with boundary conditions with the optimization conditions. The optimal control system can be written as:

71 $$\begin{aligned} \begin{aligned} \ \left\{ \begin{array}{l} \begin{array}{*{20}{l}} {dS(t)}&{} = &{}{\left[ \begin{array}{l} B - {\alpha _2}SA - \mu S\\ - {\alpha _1}\min \left\{ {1,\max \left\{ {0,\frac{{({\delta _1} - {\delta _2}){\alpha _1}SP}}{{{c_1}}}} \right\} } \right\} SP \end{array} \right] dt - {\alpha _1}{\sigma _1}SPd{W_1}(t),}\\ {dP(t)}&{} = &{}\begin{array}{l} \left[ \begin{array}{l} {\alpha _1}\min \left\{ {1,\max \left\{ {0,\frac{{({\delta _1} - {\delta _2}){\alpha _1}SP}}{{{c_1}}}} \right\} } \right\} SP\\ + \beta \min \left\{ {1,\max \left\{ {0,\frac{{({\delta _3} - {\delta _2})\beta AP}}{{{c_2}}}} \right\} } \right\} AP\\ - \min \left\{ {1,\max \left\{ {0,\frac{{({\delta _2} - {\delta _4})P}}{{{c_3}}}} \right\} } \right\} P - {\gamma _1}P - \mu P \end{array} \right] dt\\ + {\alpha _1}{\sigma _1}SPd{W_1}(t) + \beta {\sigma _2}APd{W_2}(t) - {\sigma _3}Pd{W_3}(t), \end{array}\\ {dA(t)}&{} = &{}\begin{array}{l} \left[ \begin{array}{l} {\alpha _2}SA - \beta \min \left\{ {1,\max \left\{ {0,\frac{{({\delta _3} - {\delta _2})\beta AP}}{{{c_2}}}} \right\} } \right\} AP\\ - \min \left\{ {1,\max \left\{ {0,\frac{{({\delta _3} - {\delta _5})A}}{{{c_4}}}} \right\} } \right\} A - {\gamma _2}A - \mu A \end{array} \right] dt\\ - \beta {\sigma _2}APd{W_2}(t) - {\sigma _4}Ad{W_4}(t), \end{array}\\ {d{G_1}(t)}&{} = &{}{\left[ {\min \left\{ {1,\max \left\{ {0,\frac{{({\delta _2} - {\delta _4})P}}{{{c_3}}}} \right\} } \right\} P - {\varepsilon _1}{G_1} - \mu {G_1}} \right] dt + {\sigma _3}Pd{W_3}(t),}\\ {d{G_2}(t)}&{} = &{}{\left[ {\min \left\{ {1,\max \left\{ {0,\frac{{({\delta _3} - {\delta _5})A}}{{{c_4}}}} \right\} } \right\} A - {\varepsilon _2}{G_2} - \mu {G_2}} \right] dt + {\sigma _4}Ad{W_4}(t),} \end{array}\\ \begin{array}{*{20}{l}} {\frac{{d{\delta _1}}}{{dt}}}&{} = &{}{\left[ \begin{array}{l} \left( {{\delta _1} - {\delta _2}} \right) {\alpha _1}\min \left\{ {1,\max \left\{ {0,\frac{{({\delta _1} - {\delta _2}){\alpha _1}SP}}{{{c_1}}}} \right\} } \right\} P + \left( {{\delta _1} - {\delta _3}} \right) {\alpha _2}A\\ + {\delta _1}\mu + ({\zeta _1} - {\zeta _2}){\alpha _1}{\sigma _1}P \end{array} \right] dt - {\zeta _1}d{W_1},}\\ {\frac{{d{\delta _2}}}{{dt}}}&{} = &{}{\left[ \begin{array}{l} 1 + \left( {{\delta _1} - {\delta _2}} \right) {\alpha _1}\min \left\{ {1,\max \left\{ {0,\frac{{({\delta _1} - {\delta _2}){\alpha _1}SP}}{{{c_1}}}} \right\} } \right\} S\\ + \left( {{\delta _3} - {\delta _2}} \right) \beta \min \left\{ {1,\max \left\{ {0,\frac{{({\delta _3} - {\delta _2})\beta AP}}{{{c_2}}}} \right\} } \right\} A\\ + \left( {{\delta _2} - {\delta _4}} \right) \min \left\{ {1,\max \left\{ {0,\frac{{({\delta _2} - {\delta _4})P}}{{{c_3}}}} \right\} } \right\} \\ + \left( {{\delta _2} - {\delta _2}} \right) {\gamma _1} + {\delta _2}\mu - {\zeta _4}{\sigma _3}\\ + ({\zeta _1} - {\zeta _2}){\alpha _1}{\sigma _1}S + ({\zeta _3} - {\zeta _2})\beta {\sigma _2}A + {\zeta _2}{\sigma _3}\\ \end{array} \right] dt + {\zeta _2}d{W_1} + {\zeta _2}d{W_2} - {\zeta _2}d{W_3},}\\ {\frac{{d{\delta _3}}}{{dt}}}&{} = &{}{\left[ \begin{array}{l} \left( {{\delta _1} - {\delta _3}} \right) {\alpha _2}S + \left( {{\delta _3} - {\delta _2}} \right) \beta \min \left\{ {1,\max \left\{ {0,\frac{{({\delta _3} - {\delta _2})\beta AP}}{{{c_2}}}} \right\} } \right\} P\\ + \left( {{\delta _3} - {\delta _5}} \right) {\lambda _2}(t) + \left( {{\delta _3} - {\delta _5}} \right) {\gamma _2} + {\delta _3}\mu \\ ({\zeta _3} - {\zeta _2})\beta {\sigma _2}P + {\zeta _3}{\sigma _4} - {\zeta _5}{\sigma _4} \end{array} \right] dt - {\zeta _3}d{W_2} - {\zeta _3}d{W_4},}\\ {\frac{{d{\delta _4}}}{{dt}}}&{} = &{}{\left[ {1 + \left( {{\delta _4} - {\delta _6}} \right) {\varepsilon _1} + {\delta _4}\mu } \right] dt + {\zeta _4}d{W_3},}\\ {\frac{{d{\delta _5}}}{{dt}}}&{} = &{}{\left[ {1 + \left( {{\delta _5} - {\delta _7}} \right) {\varepsilon _2} + {\delta _5}\mu } \right] dt + {\zeta _5}d{W_4},} \end{array} \end{array} \right. \ \end{aligned} \end{aligned}$$

and

72 $$\begin{aligned} \begin{aligned} \ {\delta _1}({t_f}) = {\delta _2}({t_f}) = {\delta _3}({t_f}) = {\delta _4}({t_f}) = {\delta _5}({t_f}) = 0.\ \end{aligned} \end{aligned}$$

## Numerical simulations

This section will adopt the Rung-Kutta algorithm for numerical simulation to verify the theorem proposed by the stochastic system (2). The reason of using Rung-Kutta algorithm is that the investor sentiment contagion model constructed in this paper is an ordinary differential equation with random parameter perturbation. Choosing the Rung-Kutta algorithm can quickly and stably obtain the analytical solution of the equation. Thus, the trend of investor sentiment contagion can be observed. The advantages and applicability of the Rung-Kutta algorithm are (1) Rung-Kutta method is a numerical method for solving ordinary differential equations, including nonlinear and coupled equations. (2) Rung-Kutta method can control the error and efficiency by adjusting the step size, thus adapting to different accuracy requirements. (3) Rung-Kutta method can use embedded methods to estimate and control the error, thus improving the reliability and stability. (4) Rung-Kutta method is an explicit method, which does not need to solve linear or nonlinear equations, thus reducing the computational complexity. (5) Rung-Kutta method has a wide range of applications in natural science, engineering, physics, chemistry, biology, geology and other fields, and can be used to simulate various dynamical systems, diffusion processes, wave equations, temperature changes and other phenomena.

In most previous studies, clear stipulations on the values of parameters have been lacking. Therefore, this section will combine the range of values of the basic reproductive number $R_0$ and the fundamental conditions presented in the theorem to rationalize the parameter values in the model.

To observe the influence of random factors on investor sentiment contagion and the effects of random disturbance on the characteristics of various group changes in the deterministic model, the parameter values should meet the basic condition that investor sentiment can widely spread in the social system, i.e., the basic reproductive number $R_0>1$. Thus, the parameter value was taken as $B=1, \alpha _1=0.3, \alpha _2=0.3, \beta =0.3, \theta _1=0.3, \theta _2=0.3, \lambda _1=0.1, \lambda _2=0.1, \gamma _1=0.1, \gamma _2=0.1, \epsilon _1=0.1, \epsilon _2=0.1, \mu =0.1$.

**Figure 2:**
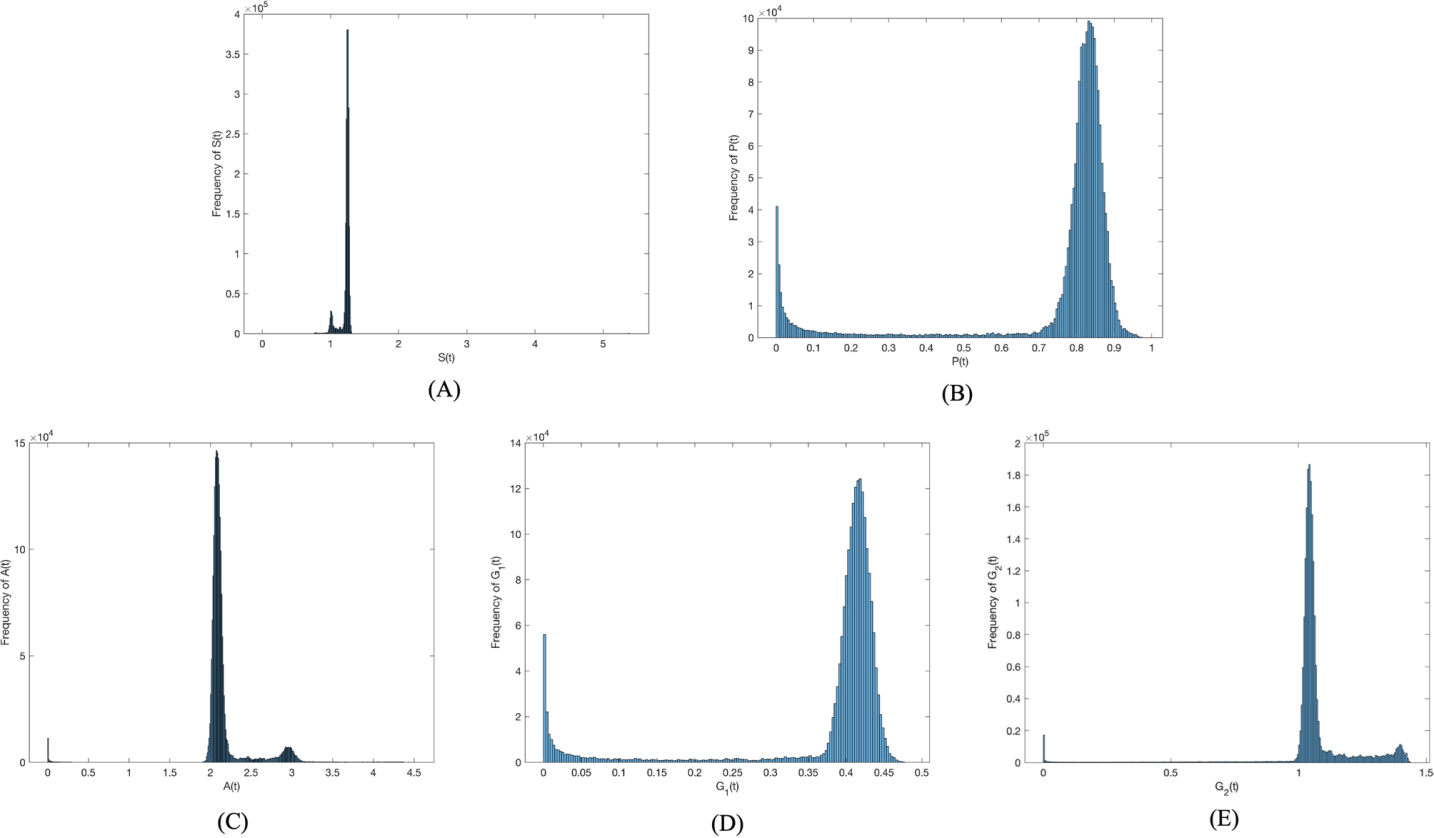
Frequency histograms of (**A**) *S*(*t*), (**B**) *P*(*t*), (**C**) *A*(*t*), (**D**) $G_1(t)$, (**E**) $G_2(t)$ when $\sigma _i(i=1,2,3,4)=0.0001$.

**Figure 3:**
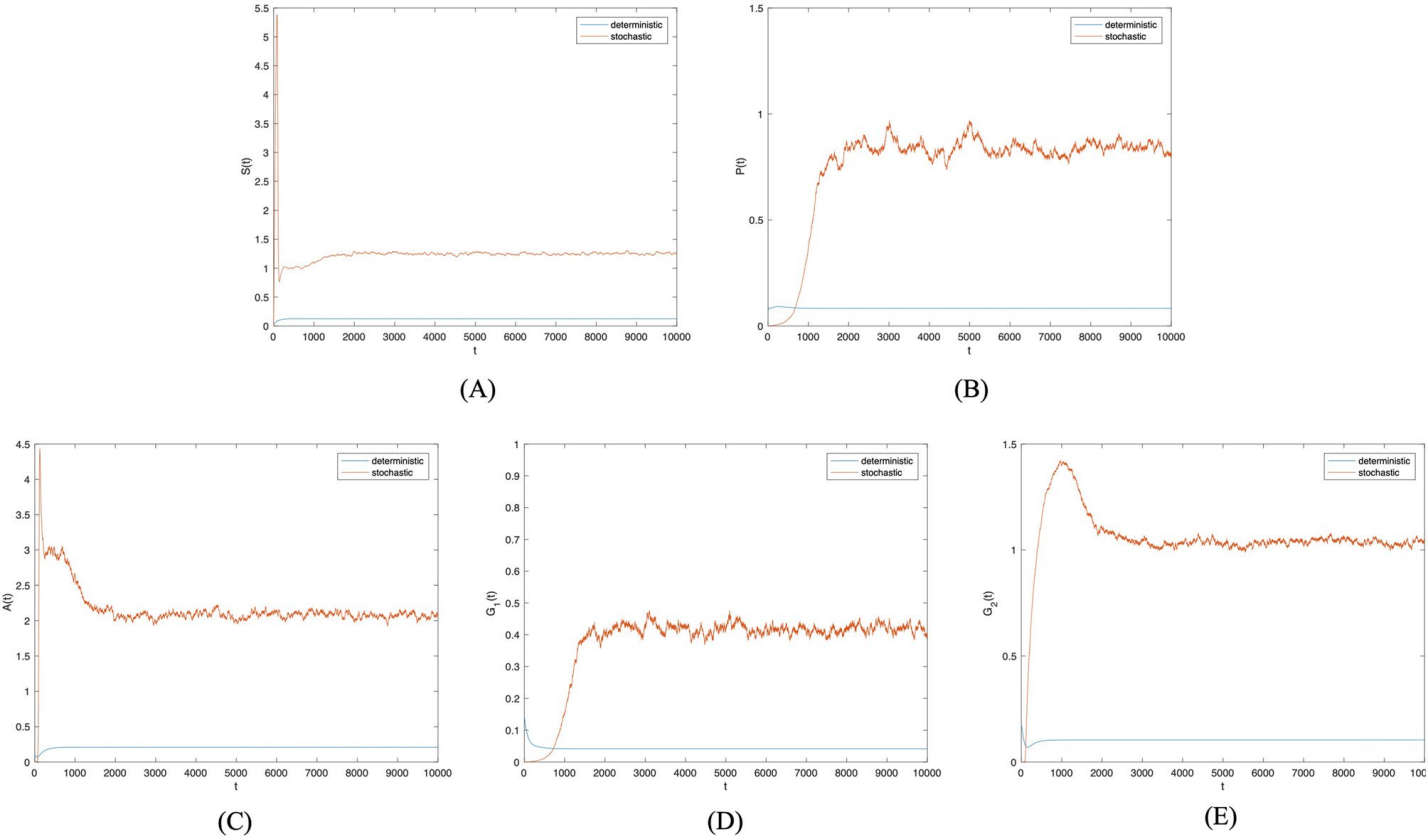
Comparison between deterministic model and stochastic model of the densities of (**A**) *S*(*t*), (**B**) *P*(*t*), (**C**) *A*(*t*), (**D**) $G_1(t)$, (**E**) $G_2(t)$ change over time when $\sigma _i(i=1,2,3,4)=0.0001$.

First, the disturbance strength $\sigma =0.0001$. Figure 2 presents the probability histogram of population $S(t), P(t), A(t), G_1(t),$ $G_2(t)$. As shown in Fig. 2, the probability of all populations adhering to the social system remains stable. Figure 3 provides a comparison of trends in population $S(t), P(t), A(t), G_1(t), G_2(t)$ between deterministic and non-deterministic systems over time. Figure 3 shows that as external random environmental factors are introduced into the social system, investor sentiment contagion in the system with random disturbance terms surpasses that in the deterministic system. This suggests a positive role played by random environmental disturbance in promoting investor sentiment contagion. Though these environmental disturbances promote investor sentiment contagion, it remains unstable in the social system, with the density of each population constantly fluctuating over time.

**Figure 4:**
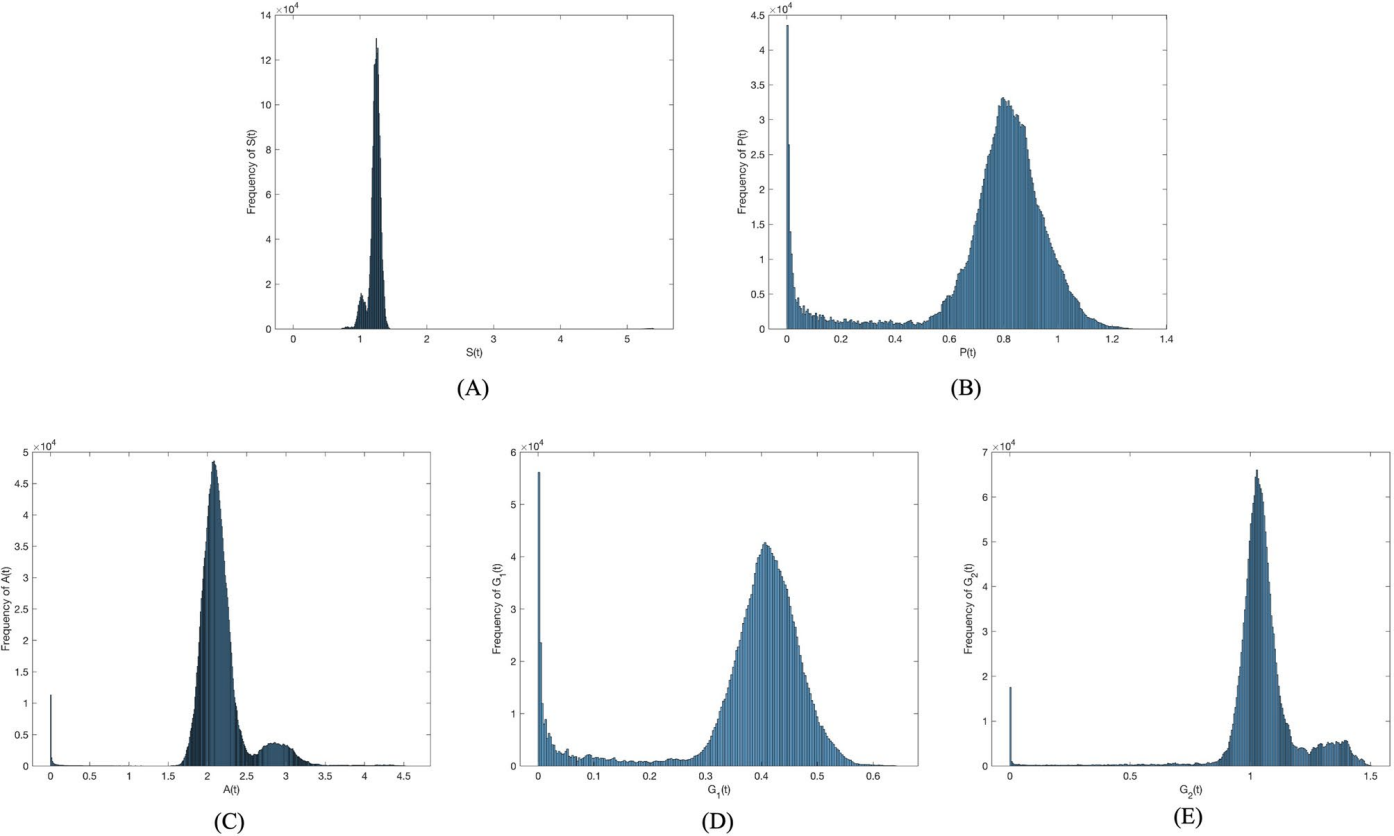
Frequency histograms of (**A**) *S*(*t*), (**B**) *P*(*t*), (**C**) *A*(*t*), (**D**) $G_1(t)$, **(E)** $G_2(t)$ when $\sigma _i(i=1,2,3,4)=0.001$.

**Figure 5:**
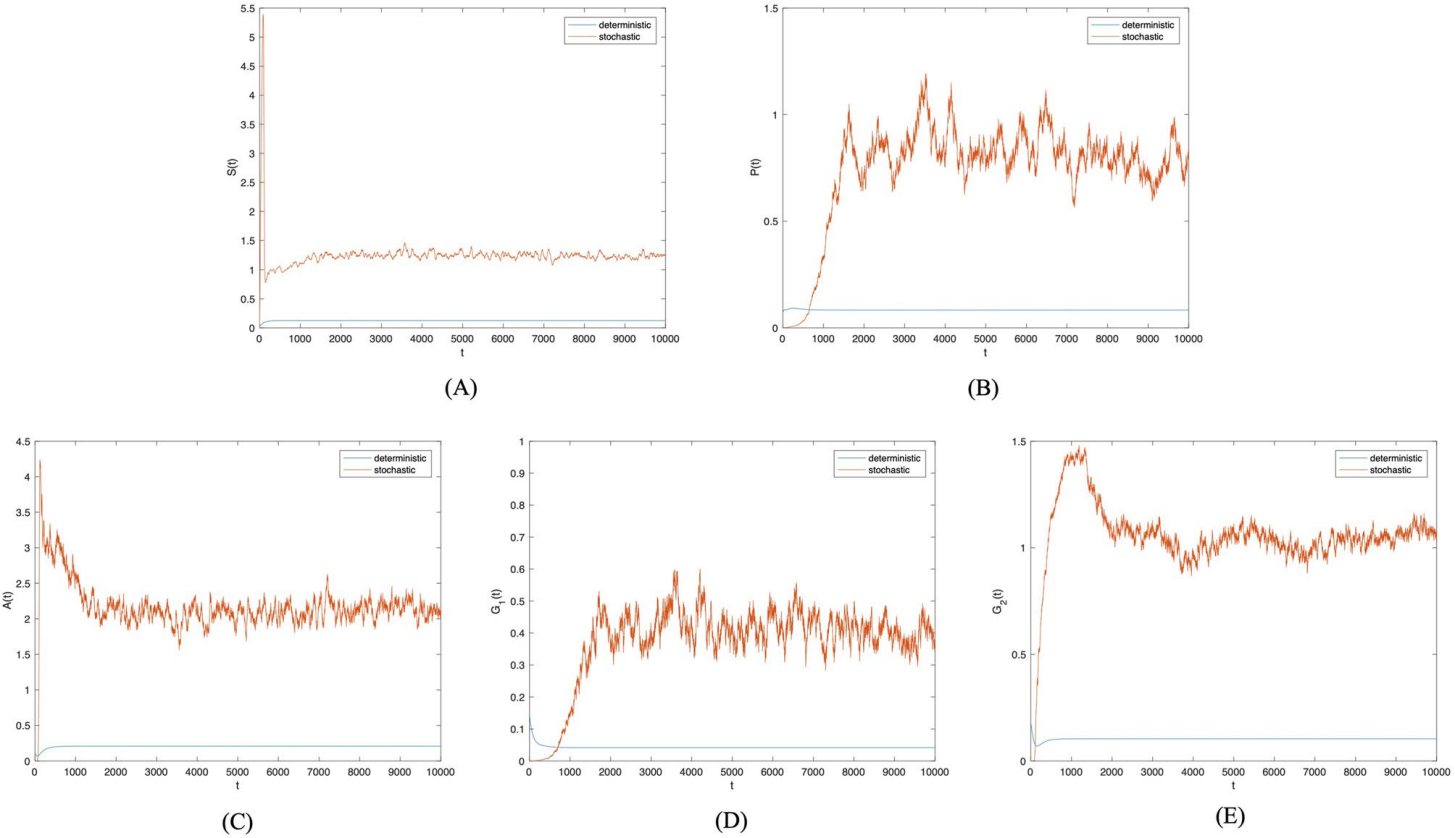
Comparison between deterministic model and stochastic model of the densities of (**A**) *S*(*t*), (**B**) *P*(*t*), (**C**) *A*(*t*), (**D**) $G_1(t)$, (**E**) $G_2(t)$ change over time when $\sigma _i(i=1,2,3,4)=0.001$.

Next, the disturbance strength was increased to $\sigma =0.001$. Figure 4 presents the probability histogram of population $S(t), P(t), A(t), G_1(t), G_2(t)$. As shown in Fig. 4, the probability of all populations adhering to the social system remains stable. Figure 5 provides a comparison of trends in population $S(t), P(t), A(t), G_1(t), G_2(t)$ between deterministic and non-deterministic systems over time. As shown in Figure 5, the increase in disturbance strength has enhanced the volatility of the system. However, the contagion trend of investor sentiment has not changed.

**Figure 6:**
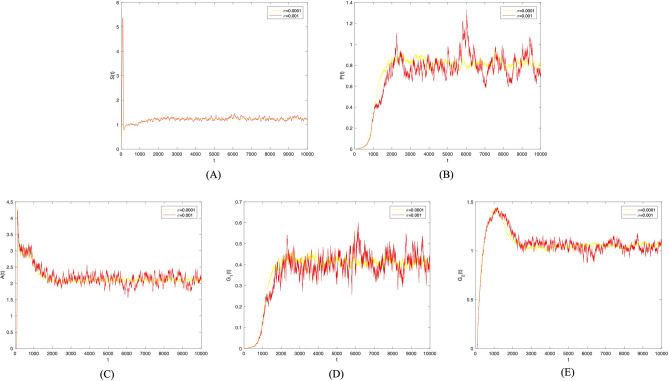
Comparison between $\sigma _i(i=1,2,3,4)=0.001$ and $\sigma _i(i=1,2,3,4)=0.0001$ of the densities of (**A**) *S*(*t*), (**B**) *P*(*t*), (**C**) *A*(*t*), (**D**) $G_1(t)$, (**E**) $G_2(t)$ change over time.

Then, to observe the impacts of different disturbance strengths on investor sentiment contagion, we combined and analyzed the trend charts of investor sentiment contagion changing over time in the non-deterministic system for disturbance strengths of 0.001 and 0.0001, respectively. As shown in Fig. 6, the fluctuation of investor sentiment contagion gradually stabilizes with the decrease in disturbance strength. This indicates that investor sentiment is more prone to spreading in a system with random environmental factors. Effectively controlling the random factors in the system can, in turn, regulate the fluctuation of investor sentiment contagion.

**Figure 7:**
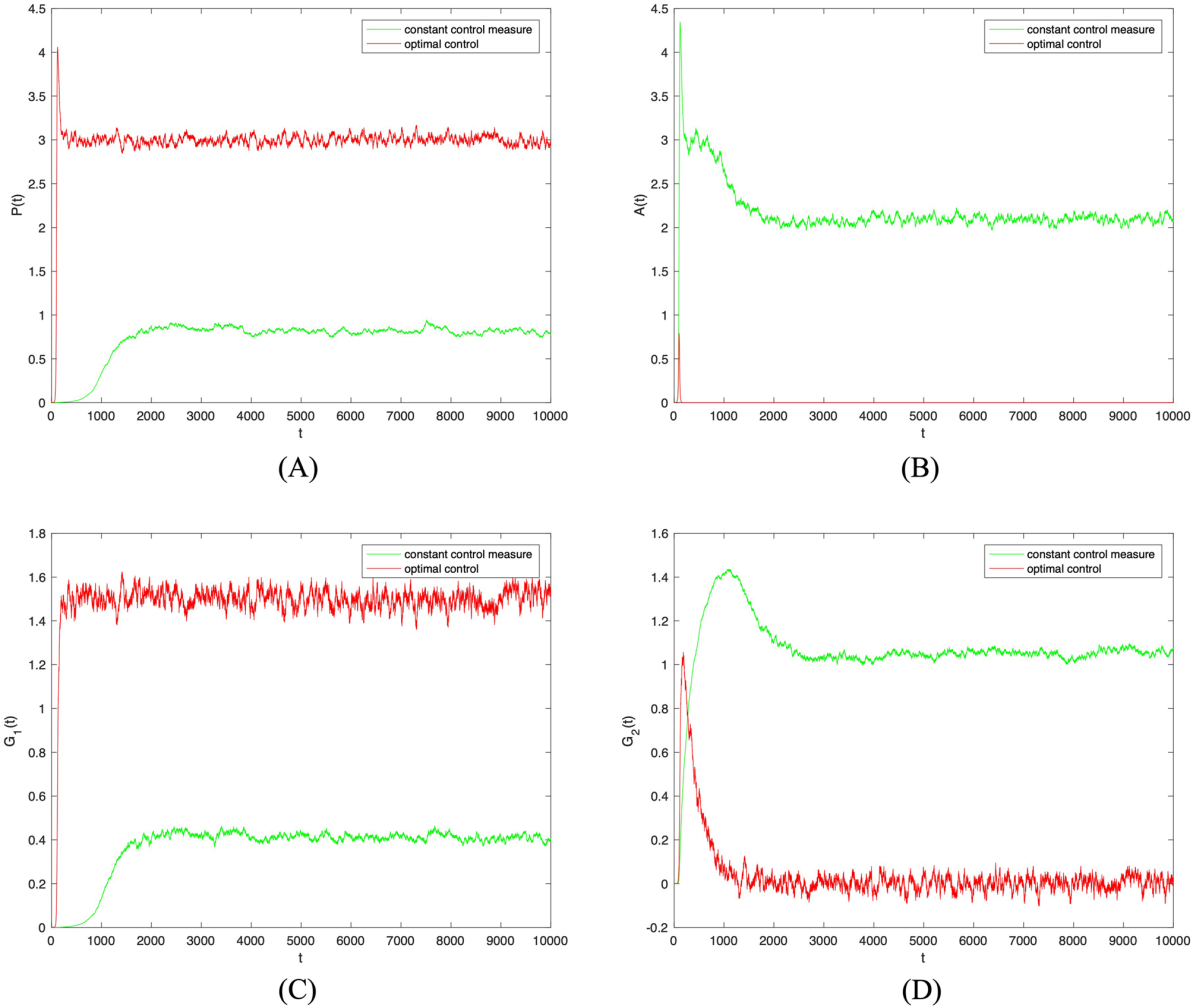
The densities of (**A**) *S*(*t*), (**B**) *P*(*t*), (**C**) *A*(*t*), (**D**) $G_1(t)$, (**E**) $G_2(t)$ change over time when $\sigma _i(i=1,2,3,4)=0.0001$ under constant control measure and optimal control.

Finally, to verify the effectiveness of the proposed control strategy, other parameters are kept constant, while random parameters $\theta _1, \theta _2, \lambda _1, \lambda _2$ are controlled. This allows observation of the trends of populations $P(t), A(t), G_1(t), G_2(t)$ changing over time when the optimal control strategy is adopted. As shown in Fig. 7, when the disturbance strength $\sigma =0.0001$ and optimal control is adopted to random parameters $\theta _1, \theta _2, \lambda _1, \lambda _2$, the densities of populations *P*(*t*) and $G_1(t)$ are superior to those without control measures. This indicates that the proposed optimal control strategy effectively promotes positive investor sentiment contagion, maximizing the regulatory isolation of investor sentiment. On the contrary, the densities of populations *A*(*t*) and $G_2(t)$ are lower than those without control measures taken. This indicates that the proposed optimal control measures can effectively curb negative investor sentiment contagion. Moreover, since negative investor sentiment is effectively controlled, additional measures to control isolated populations are unnecessary.

**Figure 8:**
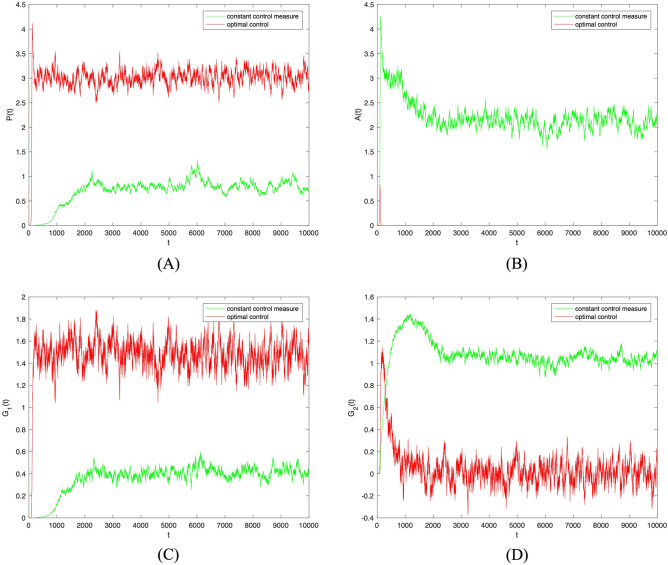
The densities of (**A**) *S*(*t*), (**B**) *P*(*t*), (**C**) *A*(*t*), (**D**) $G_1(t)$, (**E**) $G_2(t)$ change over time when $\sigma _i(i=1,2,3,4)=0.001$ under constant control measure and optimal control.

**Figure 9:**
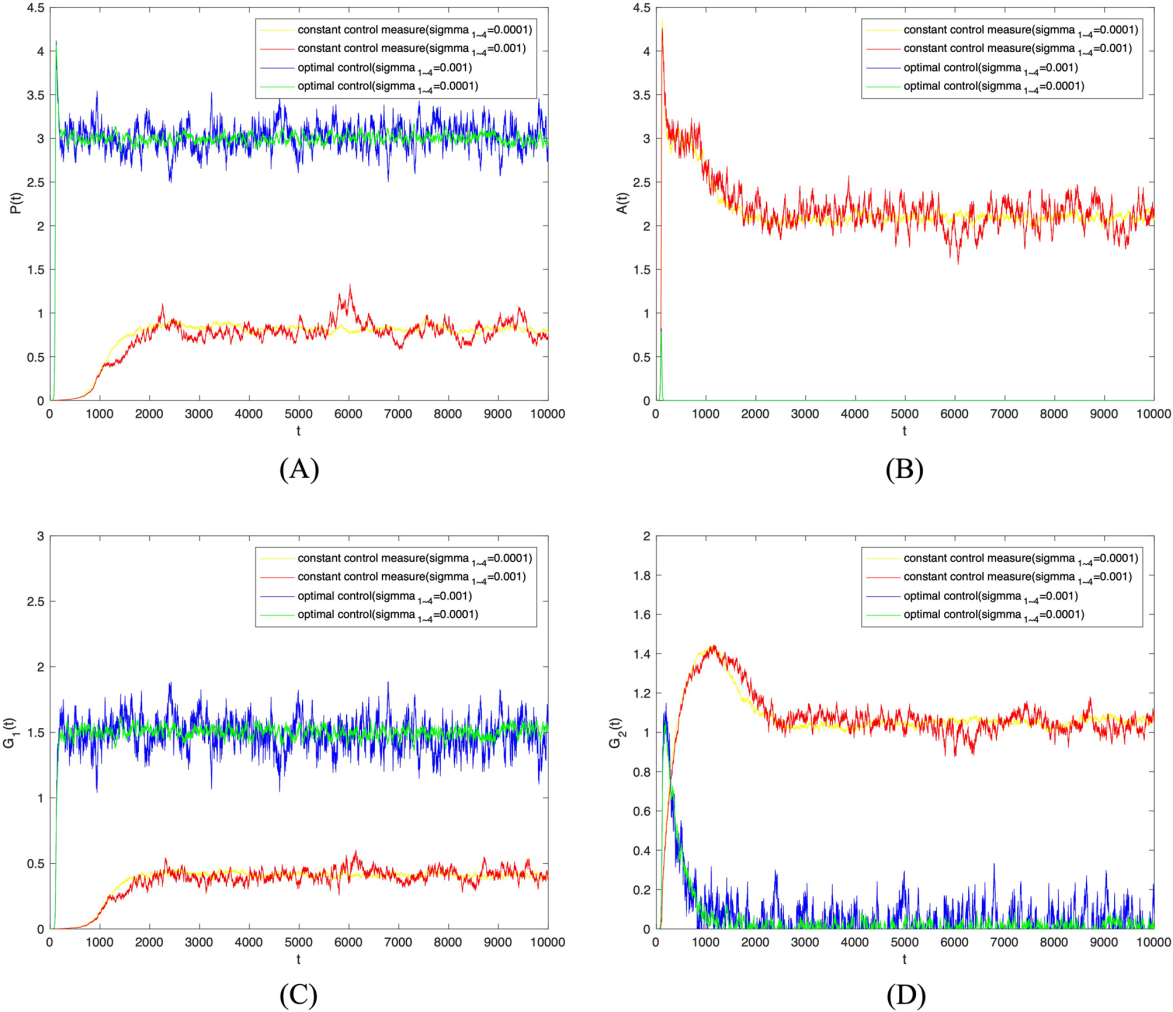
The densities of (**A**) *S*(*t*), (**B**) *P*(*t*), (**C**) *A*(*t*), (**D**) $G_1(t)$, (**E**) $G_2(t)$ change over time with different intensity of perturbation under constant control measure and optimal control.

The disturbance strength $\sigma =0.001$ was further increased. As shown in Fig. 8, when optimal control was adopted to random parameters $\theta _1, \theta _2, \lambda _1, \lambda _2$, the trend in the densities of populations $P(t), A(t), G_1(t), G_2(t)$ remains unchanged. Subsequently, the two sets of images were combined and analyzed. As shown in Fig. 9, the change of disturbance strength only affected the fluctuation of investor sentiment contagion, not the overall trend. Therefore, the optimal control strategy proposed here can effectively promote positive investor sentiment contagion and supervise investor sentiment regardless of the strength of the disturbance.

## Conclusions

In this paper, the random factors in the social system were added to the deterministic model, constructing the stochastic *SPA*2*G*2*R* model that includes parameter disturbance. Additionally, two deterministic parameters—the conversion rate of positive investor sentiment and regulatory isolation rate—were changed into non-deterministic parameters. The paper establishes the uniqueness of the global positive solution, calculates the sufficient conditions for information disappearance and stable information distribution, and presents an optimal control strategy for the stochastic model. Numerical simulations were conducted to verify the probability density distribution of the stochastic model and the influence of white noise disturbance on information transmission. Furthermore, the tendencies of information transmission under various disturbance strengths were compared.

The study yields the following results: (1) White noise disturbance has the potential to promote positive investor sentiment contagion and restrain negative investor sentiment contagion. (2) As the disturbance strength increases, the randomness of the model gradually intensifies, and the fluctuation of information transmission tendency becomes more pronounced. (3) The effective control of investor sentiment contagion can be achieved by manipulating random parameters. Notably, the optimal control strategy proposed in this study differs from previous approaches, providing the optimal value calculated based on control variables.

The approach of building a non-deterministic model of investor sentiment contagion by incorporating uncertain factors into the deterministic model aligns more closely with the complexity of the real social system. This study, based on the relevant research, uses the mean field differential equation to describe the dynamic process of investor sentiment contagion. At the same time, by introducing the random factors in the social system into the deterministic model, it can better reflect the real phenomenon of the social system. In addition, the control strategy given in this paper is based on the optimal solution calculated by the optimal control model.The research findings indicate that leveraging the randomness and complexity inherent in the economy and society can greatly promote positive investor sentiment contagion, contributing to economic and social development. For investor sentiment that is deemed unnecessary, the study recommends harnessing social fluctuations and implementing timely regulatory isolation measures.

Different from previous studies, the highlights of this article are (1) In terms of research perspective, this article used the mean field differential equation model to describe the contagion mechanism of investor sentiment, which can describe the contagion trend of investor sentiment from a microscopic perspective. (2) In terms of research methods, this article used white noise perturbation to characterize the random phenomena of social systems, and adds random parameter perturbation terms to the deterministic investor sentiment contagion model. This making the model constructed in this article more practical. (3) In terms of research results, the optimal control strategy proposed in this study differs from previous approaches, providing the optimal value calculated based on control variables. The research results of this article are different from past studies, as multiple investor sentiment exhibit a mutually inhibitory relationship during the contagion process. In addition, the control method proposed in this article can effectively promote the contagion of different investor sentiment by adjusting the random disturbance term. At the same time, the isolation of investor sentiment can quickly eliminate the contagion of various investor sentiment.

In this paper, the white noise perturbation has been used to characterize the impact of random factors in social systems on the investor sentiment contagion. And a stochastic SPA2G2R model considering different investor sentiment contagion and regulatory isolation has been constructed. White noise can clearly characterize the continuous random perturbation to the system disturbance. However, in the real social systems, the non-continuous random perturbations are also relatively common phenomena. This paper mainly focused on the impact of continuous random perturbations on the contagion of investor sentiment, without considering the impact of non-continuous random perturbations on the contagion of investor sentiment. In future research, the non-continuous random perturbation phenomena existing in social systems will be considered. And construct an investor sentiment contagion model with non-continuous random perturbations. At the same time, the L$\acute{e}$vy jump will be used to characterize the impact of non-continuous random perturbations on the contagion of investor sentiment. On this basis, the contagion trends of continuous and non-continuous random perturbations will be compared. And the different impacts of continuous and non-continuous random perturbations on the contagion of investor sentiment will be analyzed.

## Data Availability

All raw data are within the manuscript.
